# A Guide to Polysaccharide-Based Hydrogel Bioinks for 3D Bioprinting Applications

**DOI:** 10.3390/ijms23126564

**Published:** 2022-06-12

**Authors:** Maria C. Teixeira, Nicole S. Lameirinhas, João P. F. Carvalho, Armando J. D. Silvestre, Carla Vilela, Carmen S. R. Freire

**Affiliations:** CICECO—Aveiro Institute of Materials, Department of Chemistry, University of Aveiro, 3810-193 Aveiro, Portugal; maria.teixeira@ua.pt (M.C.T.); nicoleslameirinhas@ua.pt (N.S.L.); joao.pedro.carvalho@ua.pt (J.P.F.C.); armsil@ua.pt (A.J.D.S.); cvilela@ua.pt (C.V.)

**Keywords:** 3D bioprinting, bioinks, cell-laden constructs, hydrogels, polysaccharides

## Abstract

Three-dimensional (3D) bioprinting is an innovative technology in the biomedical field, allowing the fabrication of living constructs through an approach of layer-by-layer deposition of cell-laden inks, the so-called bioinks. An ideal bioink should possess proper mechanical, rheological, chemical, and biological characteristics to ensure high cell viability and the production of tissue constructs with dimensional stability and shape fidelity. Among the several types of bioinks, hydrogels are extremely appealing as they have many similarities with the extracellular matrix, providing a highly hydrated environment for cell proliferation and tunability in terms of mechanical and rheological properties. Hydrogels derived from natural polymers, and polysaccharides, in particular, are an excellent platform to mimic the extracellular matrix, given their low cytotoxicity, high hydrophilicity, and diversity of structures. In fact, polysaccharide-based hydrogels are trendy materials for 3D bioprinting since they are abundant and combine adequate physicochemical and biomimetic features for the development of novel bioinks. Thus, this review portrays the most relevant advances in polysaccharide-based hydrogel bioinks for 3D bioprinting, focusing on the last five years, with emphasis on their properties, advantages, and limitations, considering polysaccharide families classified according to their source, namely from seaweed, higher plants, microbial, and animal (particularly crustaceans) origin.

## 1. Introduction

Three-dimensional (3D) bioprinting technology is an innovative and promising strategy for engineering 3D living tissue constructs with well-defined structures and geometries [1]. The principle of 3D bioprinting involves computer-aided design for the controlled manufacture of 3D living structures in a layer-by-layer approach [2]. The bioprinting of cell-laden biomaterials, viz. the bioinks, is achieved through several techniques, including extrusion printing [3], inkjet printing [4], laser-assisted printing [5], and stereolithography [6]. Currently, 3D bioprinting technology has received immense attention and is widely investigated for broad-spectrum biomedical applications [7], such as tissue engineering and transplantation [8], drugs screening [9], and cancer research [10].

The engineering of novel bioink formulations with adequate properties is a major area of research because of their key role in the success of the bioprinting process. The design and optimization of bioinks aim to explore and manipulate artificial biological and biochemical environments that could accommodate and allow the growth of living cells in combination with suitable rheological and mechanical properties [11]. After printing, the 3D constructs are expected to keep their pre-designed shape and physical integrity for a defined period of time while maintaining cell viability and proliferation ability [12,13]. Thus, the key features of an ideal bioink are related to biocompatibility and biodegradability, high mechanical integrity and stability, and the ability to promote cell adhesion and proliferation [14].

Bioinks typically consist of biomaterials, cells, bioactive molecules (e.g., growth factors), and their combinations [15] and are commonly divided into two major types, namely scaffold-based bioinks, where the cells are loaded in an exogenous material (i.e., hydrogels, microcarriers, and decellularized matrix components) and then printed [16], or scaffold-free bioinks, where the cells are first assembled into neo-tissues (i.e., cell aggregates, tissue strands, and tissue spheroids) with suitable properties for bioprinting and which, after deposition, mature and evolve into functional living tissues [17]. Among all of them, hydrogel-based bioinks are the most described and investigated biomaterials [18].

Hydrogels (either from natural or synthetic origin) refer to a class of crosslinked polymeric materials capable of absorbing and retaining large quantities of water [19]. Therefore, hydrogels consist in highly hydrated 3D environments, very similar to the extracellular matrix (ECM). Moreover, their unique architecture provides permeability to oxygen, nutrients, and other water-soluble compounds, in addition to allowing cellular migration and communication through their porous flexible network [19]. Hydrogels can be obtained using different crosslinking approaches, including chemical (establishment of covalent bonds by chemical reactions) and physical (induced gelation by ionic, thermal, or pH stimuli) strategies [20]. Natural polymers, also commonly referred to as biopolymers, include macromolecules such as polysaccharides (e.g., cellulose, chitin (and its derivative, chitosan), alginic acid (and in particular its salt form, alginate), pectin, hyaluronic acid, and starch) and proteins (e.g., collagen and fibrin), biosynthesized by living organisms, including plants, animals, algae, bacteria, and fungi [21,22,23]. The use of biopolymeric hydrogel-based bioinks for 3D bioprinting applications offers inherent advantages, as their biocompatibility toward mammalian cells and tissues, and most biopolymers are biodegraded under physiological conditions, with the formation of nontoxic degradation products [24]. Moreover, biopolymers are classified as eco-friendly materials due to their renewable and biodegradable natures [22]. In turn, the main weakness of biopolymers is the difficulty in obtaining materials with reproducible quality and properties, since these heavily depend on the biopolymers source, which often leads to high batch-to-batch variations [22,25] and, thus, some lack of reproducibility in their processing into final materials.

Among the panoply of biopolymers with the ability to form hydrogels, polysaccharides and their derivatives are increasingly popular for 3D bioprinting applications and particularly for the engineering and development of bioink formulations [26]. This attractiveness relies on their main features, including ease of derivatization/functionalization, high diversity of chemical structures, adequate rheological and mechanical properties, and intrinsic biocompatibility and biodegradability. In fact, some polysaccharides (e.g., glycosaminoglycans) are present in the ECM and, in general, are molecularly similar to other ECM components, namely, glycoproteins and glycolipids [27]. Therefore, most polysaccharide-based hydrogels are biomaterials adequate for cell encapsulation, since they are typically prepared using aqueous systems and mild crosslinking methods, following diverse physical and chemical approaches that allow the control of the mechanical integrity, morphology, and gel properties [28].

The developments in the field of bioinks for 3D bioprinting applications have been the theme of several appraisals [18,29,30,31,32,33,34,35,36,37,38,39,40,41,42,43,44,45,46,47,48,49,50,51,52]. Specifically, the progress over the last two decades was reviewed by Pedroza-Gonzalez et al. [39], who provided a systematic analysis of more than 390 original papers from 2000 to 2019. From a materials perspective, many of these revisions gave special emphasis to hydrogel bioinks [18,35,36,50,52,53] and to natural materials-based bioinks [33,38]. Moreover, recent reviews have also focused on the use of specific polysaccharides to produce bioinks, namely alginate [29,33,37,38], (nano)cellulose [32,35,40,41,46,47,49], chitosan [44], and hyaluronic acid [31]. Most recently, Mahendiran et al. [37] provided an overview of 3D printing technologies and the use of different plant-based bioinks in tissue engineering, focusing on plant polysaccharides of terrestrial (starch, nanocellulose, and pectin) and marine (ulvan, sodium alginate (commonly referred as alginate), fucoidan, agarose, and carrageenan) origins [37]. Nonetheless, a comprehensive compendium on the distinct polysaccharide families (from different origins) to develop hydrogel-based bioinks has never been reported.

**Figure 1:**
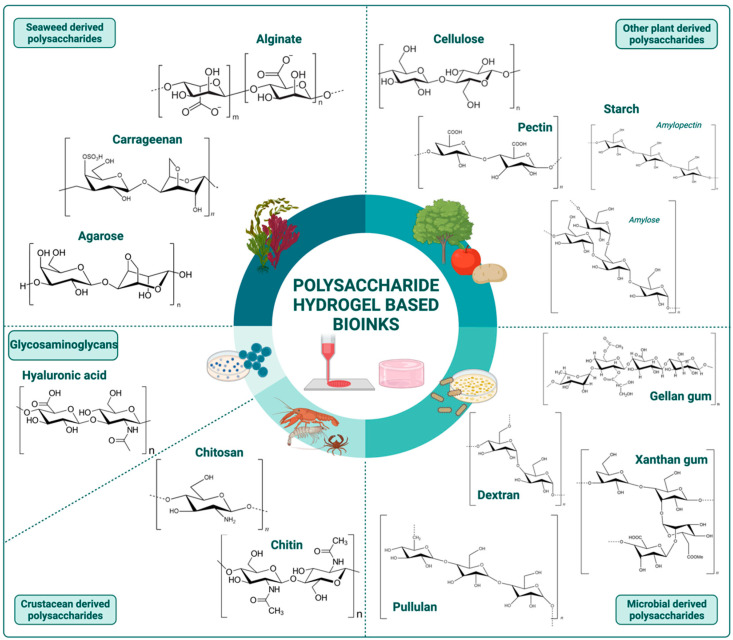
Most relevant polysaccharides from different sources used as raw materials for the fabrication of hydrogel-based bioinks for 3D bioprinting applications. Image created with BioRender.com.

In this context, the present review describes a collection of information about the use of polysaccharide-based hydrogels as bioinks for 3D bioprinting. The suitability and versatility of these natural polymers for the development of hydrogel bioink formulations are presented, considering the most representative polysaccharides obtained from different sources, namely from seaweed (alginate, carrageenan, and agarose), other plants viz. higher plants (cellulose, pectin, and starch), microbial origin (dextran, xanthan gum, gellan gum, and pullulan), and animal sources, particularly from crustaceans (chitin and chitosan), as depicted in Figure 1. Glycosaminoglycans, because of their specific properties and diverse origins (plants, animals, and microbial), are considered in a separate section. The polysaccharides’ advantages versus limitations, and strategies to overcome them, are highlighted and supported by the most recent advances in their use for the development of bioinks for the 3D bioprinting of various types of living tissue structures. Finally, the main challenges and future perspectives on this matter are discussed.

## 2. Polysaccharide-Based Hydrogel Bioinks

### 2.1. Seaweed Derived Polysaccharides

Among marine resources, algae, also referred as seaweed, are a well-known natural source of a high diversity of polysaccharides [53]. Seaweeds are classified into three main groups based on their main photosynthetic pigments, viz. red (*Rhodophyceae*), brown (*Phaeophyceae*), and green (*Chlorophyceae*) algae. Polysaccharides are the major components of seaweeds, accounting for up to 70% of their dry weights, and are mainly present on their cell walls [53,54].

Among the various seaweed-derived polysaccharides, alginic acid (and particularly its salt form, sodium alginate) [55,56,57,58,59,60,61,62,63,64,65,66], carrageenan [67,68,69,70,71,72,73,74], and agarose [75,76,77,78,79] have been widely used as polymeric matrices (either solely or in combination with other polysaccharides or proteins) for the development of hydrogel-based bioinks for 3D bioprinting, as outlined in Table 1.

#### 2.1.1. Alginate

Alginate is a polyanionic water-soluble linear polysaccharide extracted from brown algae. Alginate polymeric chains consist of mannuronate(M) and guluronate(G) units arranged in different proportions and motif blocks and have a molecular weight that can range from 10 kDa to 600 kDa, depending on the algae source [80]. Alginate forms hydrogels under mild conditions, almost instantaneously, by ionotropic gelation with divalent cations, such as Ca^2+^ [81]. This process follows the “egg-box” model where Ca^2+^ ions are entrapped within cavities formed by a cooperative coupling of contiguous G units. Thus, apart from polymer concentration and molecular weight, G unit content is also an important parameter that influences relevant alginate hydrogel properties, such as viscosity, elasticity, and pore size [82].

Indeed, Ca^2+^-alginate hydrogels are one of the most studied systems in the field of 3D bioprinting (Table 1) due to their excellent tunability and printability, as reflected and documented in the dedicated literature reviews about the main progress in the domain of alginate-based bioinks since 2016 [29,33,37,38]. Despite its widely recognized and explored advantages, some recent research efforts on the development of alginate-based bioinks are still focused on tackling the biological and mechanical limitations of alginate hydrogels. Specifically, alginate is a relatively inert biopolymer, lacking cell-binding receptors, which does not favor cell adhesion and proliferation in alginate-based bioinks [25]. Additionally, due to some degree of unpredictability in their degradation rates, 3D bioprinted alginate-based tissue constructs often lack long-term mechanical stability [83].

In order to address the inertness of alginate, recent studies continue to explore the chemical modification of alginate hydrogels by engrafting cell-adhesive peptides, such as Arg-Gly-Asp (RGD) and Tyr-Ile-Gly-Ser-Arg (YIGSR) moieties, a strategy widely adopted in the production of biomimetic scaffolds for tissue engineering [84]. For example, following this approach, Sarker et al. [60], developed alginate bioinks for neural tissue engineering. In this study, alginate was conjugated with both RGD and YIGSR peptides using carbodiimide to produce Schwann cells-laden bioinks with improved biological properties. In fact, the simultaneous chemical engrafting of both peptides in alginate at 2% (*w*/*v*) promoted high cell viability (~95%) for up to 7 days [60]. Despite the good results obtained with this study, it is important to underline that these approaches are typically very expensive due to the high production costs and low production yields of these peptide binding motifs.

Other strategies have been explored to improve cell proliferation on alginate-based hydrogels, namely by combining the latter with bioactive materials [63,64]. As an example, Liu et al. [63] developed a bioink by blending alginate with albumen, commonly known as egg white, a protein-rich biomaterial, and umbilical vein endothelial cells (HUVECs). The cell viability, evaluated by the cell optical densities of the bioinks composed of alginate at 5% (*w*/*v*) with albumen, in a ratio of 5:1, was considerably higher compared to the cell-laden alginate hydrogels after 5 days [63]. This combination was also explored by Delkash et al. [63], who prepared bioinks by directly dissolving alginate in pasteurized egg white in different concentrations (1, 1.5, 2, 2.5, and 3% (*w*/*v*)) and then loaded the mixture with HUVECs. Rheological characterization of the printed constructs obtained by extrusion printing (with alginate concentrations of 2 and 3% (*w*/*v*) selected for the printing tests) revealed a storage modulus (G’) varying between 20 to 27 kPa, which is similar to those of heart tissue samples. The cell viability of HUVECs determined by LIVE/DEAD assays was about 94%, 7 days after printing [64].

**Figure 2:**
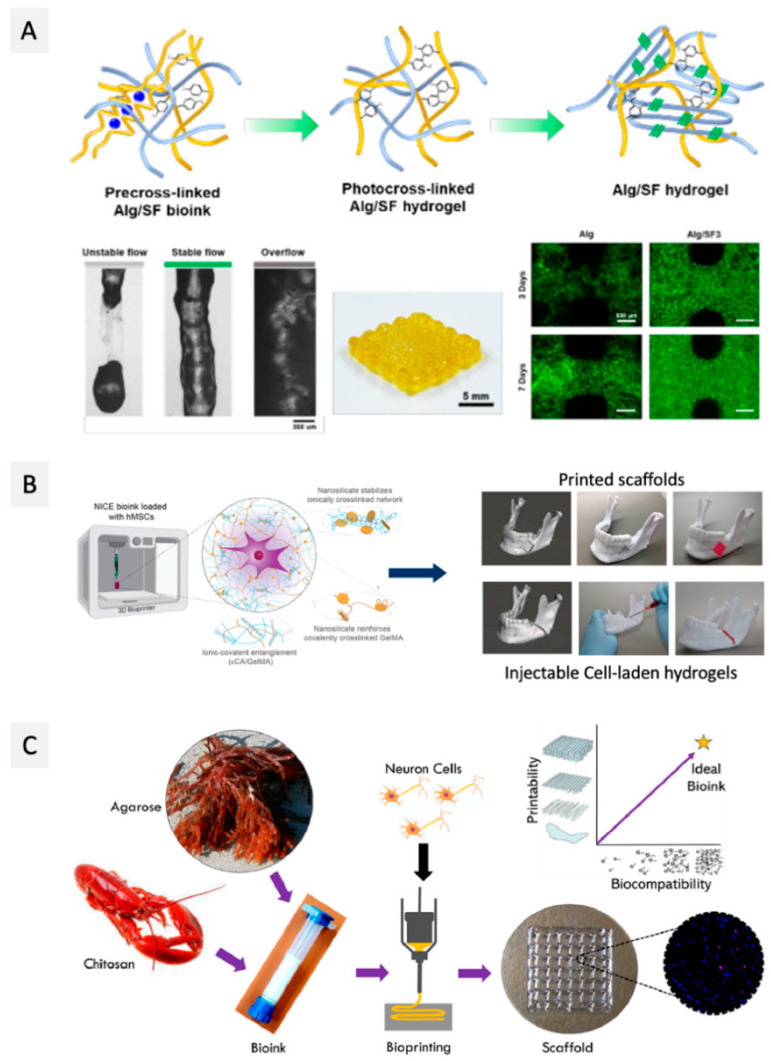
Schematic representations of some approaches used for the manufacturing of bioinks from seaweed derived polysaccharides. (**A**) Alginate/SFMA bioinks pre-crosslinked with CaCO_3_ and then photocrosslinked to produce stable printed constructs with high cell viability (Reproduced with permission from [66]. Copyright American Chemical Society, 2021); (**B**) NICE bioinks composed by carrageenan, nSi, and GelMA to produce printed mandibular models (Reproduced with permission from [74]. Copyright American Chemical Society, 2020); and (**C**) bioinks combining agarose and NOOC with high printability and cell viability (Reproduced with permission from [79]. Copyright Elsevier, 2021).

The combination of alginate with nanofibrillar bio-based materials such as protein-based nanofibers has also been used with the same purpose, with a special focus on silk fibroin (SF), a natural biomaterial extracted from *Bombyx mori* silkworms, known for its excellent biological properties and good mechanical performance [61,66]. As an illustration, Li et al. [61] explored the combination of SF fibers with alginate and Pluronic F127 (used as a sacrificial polymer) to produce a vascularized liver mimetic tissue. The addition of 5% (*w*/*v*) of SF to the bioink formulation of alginate (5% (*w*/*v*)) and Pluronic F127 (13% (*w*/*v*)) not only promoted a 99.5% cell viability of liver cancer cells (C3A) 14 days after printing, but the printed scaffolds of alginate and SF also showed improved mechanical properties, with compressive modulus increasing from 11.5 ± 1.6 kPa (for alginate) to 16.0 ± 2.5 kPa (for alginate/SF) [61]. In a more recent study, Kim et al. [66] designed a bioink composed of alginate (3 wt.%) and SF methacrylate (SFMA) (1, 3 and 5 wt.%) loaded with NIH-3T3 fibroblasts, taking advantage of two different crosslinking methods (ionic gelation for alginate and UV crosslinking for SFMA). The production of the printed scaffolds comprised two steps: First, the alginate/SFMA bioinks were pre-crosslinked with CaCO_3_ to reach proper viscosity for extrusion printing, and second, the printed constructs were crosslinked by UV light. The cells’ viability was kept close to 95% up to 7 days after printing, and SF promoted cell proliferation (Figure 2A), as demonstrated by the increased optical density for the printed constructs [66].

The production of alginate nanocomposites using other biobased reinforcing nanostructures has also attracted enormous attention. In fact, nanocellulose fibers (and other cellulose-based nanostructures, such as cellulose nanocrystals) have been widely used as reinforcement additives for alginate hydrogels, as will be discussed below (Section 2.2.1, dedicated to cellulose-based bioinks).

Due to the proven and cumulated knowledge regarding alginate hydrogels bioinks in the latter years, the most important efforts on the development of novel alginate-based bioinks have been essentially centered on creating more sophisticated 3D living scaffolds from alginate that allow the fabrication of both “soft” (skin) [57] and “hard” (bone and cartilage) [55,56] biomimetic tissue constructs, on engineering vascular structures [62,65] and on the design of disease models [58,59]. On this matter, for instance, Somasekharan et al. [57] reported a bioink based on alginate blended with gelatin and diethylaminoethyl cellulose (DCEL) to produce skin tissue analogues by extrusion bioprinting. The addition of gelatin enhanced the cell adhesion due to the intrinsic presence of RGD peptide sequences on the gelatin backbone, and the incorporation of DCEL provided matrix stability and improved mechanical properties because of its fibrous nature. The optimal formulation composed of alginate 2% (*w*/*v*), gelatin 3.3% (*w*/*v*), and DCEL 0.93% (*w*/*v*), was loaded with a dual cell culture of primary adult fibroblasts and primary epidermal keratinocytes, and the bioprinted scaffolds showed suitable mechanical properties when compared to skin tissue, namely elasticity, with a Young’s modulus of 125 ± 22 kPa and elongation of break of 91.7 ± 9.36%. Fibroblasts and keratinocytes were co-cultured, and cell differentiation within the scaffolds was monitored for 21 days. At this point in time, histological analysis showed the formation of both dermal and epidermal equivalent structures [57].

Another important challenge in the field of bioprinting is the production of vascularized structures to enhance cell nutrient delivery and oxygen perfusion [85]. In this realm, Li et al. [62] developed an alginate/gelatin/carbon nanotubes (CNTs) hybrid bioink to manufacture cylindrical scaffolds loaded with mouse epidermal fibroblasts, envisioning the engineering of artificial blood vessels. The use of a modified extrusion method allowed the production of hollow tubular scaffolds. The addition of 0.5% (*w*/*v*) of CNTs to the alginate/gelatin blend mechanically reinforced the resulting bioink with only mild toxicity to fibroblasts, which were maintained viable until 7 days, with a cell survival rate of ~85% [62].

In a more recent study, Dogan et al. [65] took a step further by producing bioprinted scaffolds using an alginate-collagen I bioink loaded with human induced pluripotent stem cell-derived mesodermal progenitor cells (hiMPCs), cultured with the addition of vascular endothelial growth factor (VEGF) to induce the formation of blood vessels. After 21 days, it was possible to observe the formation of small and large vessels within the bioprinted scaffolds, which were then transplanted into a chicken embryo chorioallantoic membrane (CAM) to test their functionality. The printed vessels allowed proper blood perfusion within the CAM model [65].

The development of advanced disease models, based on organ-on-chip technologies, to study disease mechanisms and explore new drugs is also an important contribution of the 3D bioprinting technology. For instance, Lewiki et al. [59] developed a bioprinted neuroblastoma model. Specifically, this study aimed at the optimization of the precise printing parameters to maximize cell viability in alginate hydrogels at 2% (*w*/*v*) for 7 days, creating a tumor model for the testing of novel anti-tumoral drugs [59]. Following the same premise, Schmid et al. developed an alginate bioink with hyaluronic acid and gelatin (0.5% alginate, 0.1% hyaluronic acid, 3% gelatin (*w*/*v*)) loaded with malignant melanoma cells (Mel Im) to produce a melanoma 3D disease model [58].

#### 2.1.2. Carrageenan

Carrageenan is an anionic sulfated polysaccharide extracted from the *Rhodophyceae* red algae that occurs in six different forms, depending on their sulfate content, source, and water-solubility, viz. (Kappa)κ-, (Iota)ι-, (Lambda)λ-, (Mu)µ-, (Nu)ν-, and (Theta)θ-carrageenans [86]. Among them, κ-, ι-, and λ-carrageenans are the most popular and commercially available ones, because of their excellent gelling and viscoelastic properties, with molecular weights ranging from 200 to 800 kDa [87]. Carrageenans can form hydrogels in the presence of mono- or divalent cations (e.g., K^+^ or Ca^2+^) due to the establishment of interactions of those cations with the sulfate groups. Moreover, and as for most hydrogels, the gelling process and gel viscosity of carrageenan depend on multiple parameters, including the carrageenan form and sulfate content, molecular weight, concentration, and temperature [86].

The application of carrageenan-based hydrogels in the biomedical field is well-known, particularly in drug delivery, tissue engineering, and wound healing, as reviewed by Yegappan et al. [88] and, most recently, by Jafari et al. [89], who also highlighted the potential of carrageenan hydrogels for the formulation of bioinks. Several strategies have been explored to develop carrageenan-based hydrogel bioinks with adequate properties, namely the incorporation of nanostructures, such as nanosilicates (nSi) [69,70,75], the combination with other biopolymers, such as gelatin [70,71] and alginate [73], and even the chemical modification of the carrageenan macromolecular backbone with the production of methacrylated derivatives [67,72], envisioning to improve some limitations mainly related with their rheological properties and poor mechanical stability under physiological conditions [88].

As an illustrative example, Wilson and co-workers [68] investigated the addition of nSi to a κ-carrageenan hydrogel to tune its shear-thinning and thermo-reversibility for improved printability. The addition of 6 wt.% of nSi to a 2.5% wt.% κ-carrageenan gel decreased the gelling temperature from 40 °C to 35 °C, allowing the printing of the hydrogel at physiological temperature. The addition of nSi also increased the compressive modulus of the resulting hydrogels by about 2.5-fold, reaching 210 kPa for the κ-carrageenan 2.5%-nSi 6 wt.% bioink formulation. Complex anatomical structures, such as a nose or an ear, were printed by extrusion, and the obtained constructs were crosslinked in an aqueous K^+^ salt bath, allowing the production of mechanically resilient structures. Moreover, MC3T3-E1 mouse pre-osteoblasts were incorporated into the nSi-κ-carrageenan bioink and were found to be viable 7 days after printing of the constructs, based on the evaluation of the normalized Alamar blue percentage reduction [68]. In a subsequent study [69], an ionic-covalent entangled bioink was produced by adding gelatin-methacrylate (GelMA) to the carrageenan/nSi blend. Carrageenan (1% *w*/*v*)/nSi (2% *w*/*v*)/GelMA (10% *w*/*v*) printed scaffolds were double crosslinked by UV (due to the addition of GelMA) and a K^+^ aqueous solution, displaying improved mechanical properties, namely higher stiffness, toughness, and elasticity. Moreover, compression tests revealed that single carrageenan hydrogels with only ionic crosslinking showed poor recovery properties (<30%), while the inclusion of the nSi and GelMA increased the recovery percentage up to 75%. In addition, encapsulated MC3T3-E1 cells survived (cell viability >90%) and proliferated within the constructs for up to 120 days [69]. These results motivated an additional study [74], where the carrageenan/nSi/GelMA bioink was optimized for bone tissue engineering, using human mesenchymal stem cells (hMSCs) and different cartilage and/or bone-like ECM components, including glycosaminoglycans and proteoglycans, as displayed in Figure 2B. Similar results in terms of mechanical performance and cell viability were observed for these systems [74].

Regarding the strategy of combining carrageenan with other biopolymers, for example, Kim et al. [73] blended κ-carrageenan with alginate to prepare a bioink laden with mesenchymal stem cells (MSCs) and studied its properties for extrusion-based bioprinting. The combination of alginate 2% (*w*/*v*) with different amounts of carrageenan, namely 0.5, 1, and 1.5% (*w*/*v*), resulted in bioinks with enhanced rheological behavior, specifically with increasing viscosity values with the increments on the concentration of carrageenan. Although carrageenan is not the main component of these formulations, it is used to improve important properties of the bioinks. The increasing amount of carrageenan on the bioinks formulations also resulted in improved mechanical performance, with G’ increasing up to 900 Pa. Moreover, the viability of MSCs laden on the printed constructs for up to 3 days was higher for carrageenan-alginate bioinks in comparison to the single alginate ones when evaluated by the normalized Alamar blue percentage reduction [73]. These results are certainly related to the better biological properties of carrageenan compared to those of alginate, which are known to be quite biologically inert, as previously referred.

In a different vein, Li and co-workers [70,71] developed a methodology to improve the interfacial bonding of a 3D-printed multilayered structure by taking advantage of the electrostatic interactions between two oppositely charged hydrogel inks based on κ-carrageenan and gelatin, respectively. The combination of carrageenan and gelatin overcomes the limitation of using gelatin for bioprinting at 37 °C without a further post-crosslinking step. Apart from the improvement of the mechanical stability, the viability of mouse myoblasts C2C12 cells on the printed constructs after 24 h was above 90% for the carrageenan and gelatin concentrations of 2 and 8 wt.% [71]. To further explore this approach, the authors studied different anionic (alginate, xanthan, and carrageenan) and cationic (chitosan, gelatin, and GelMA) hydrogels combinations. Based on the rheological properties of the printed hydrogels and the structural integrity of the printed constructs, it was found that the best combination was carrageenan (2 wt.%) and GelMA (10 wt.%). In this case, apart from the ionic interactions between the different layers, the UV crosslinking of the GelMA layers obviously resulted in constructs with improved structural integrity and mechanical properties. However, the UV crosslinking did not affect the myoblasts loaded in the two hydrogels bioinks since the bioprinted constructs showed cell viabilities above 96%, up to 7 days post bioprinting [70].

As a different strategy to improve the properties of carrageenan hydrogel bioinks, the chemical modification of κ-carrageenan with methacrylate groups was first reported by Mihalia et al. [67] to produce hydrogels by two consecutive crosslinking steps, namely with exposure to UV irradiation followed by treatment with K^+^. These hydrogels showed good printability for extrusion bioprinting, and the bioprinted scaffolds loaded with hMSCs presented good cell viability (∼75%) for long periods (up to 21 days) [67]. In a more recent study, methacrylated κ-carrageenan (1% *w*/*v*) was blended with GelMA (10% *w*/*v*) [72] to fabricate 3D-printed scaffolds to support adipose tissue regeneration, with a specific application in breast reconstruction. Scaffolds printed by extrusion of the bioinks seeded with adipose tissue-derived stem cells (ASCs), and crosslinked via UV radiation, showed similar mechanical properties to those of native breast tissue (Young’s modulus of 2 kPa) and were proven to be stable up to 21 days, maintaining high cell viability (>94%), and induced cell proliferation (proliferation rate > 128%) up to 14 days [72]. In fact, to the best of our knowledge, the only commercially available carrageenan-based bioink (KapMA) is composed of methacrylated carrageenan, and it is commercialized by AdBioInk [90].

#### 2.1.3. Agarose

Agarose, the gelling fraction of agar–agar, is a neutral linear polysaccharide present in the cell walls of red algae, soluble in water at 95–100 °C, with a molecular weight between 80 and 140 kDa, and high gelling strength even at very low concentrations, creating thermoreversible gels [91]. The gelling mechanism of agarose occurs upon cooling and lies in the establishment of intermolecular hydrogen bonds, which lead to the formation of side-by-side chain aggregates originating the gel network [92]. Once agarose gels are formed, at around 32–34 °C, they are stable and do not re-liquefy until heated to 65 °C [93].

Agarose hydrogels are characterized by their good mechanical properties and long-term stability [91], which are some of the requirements for bioinks development. However, the use of agarose hydrogels for bioprinting is still very limited, mainly because of their biological inertness, resulting in poor cell viability in long-term cultures [91]. In fact, to the best of our knowledge, only two studies have been reported so far [75,79]. The first, by López-Marcial et al. [75], compared the mechanical and rheological properties, including yield stress, storage modulus, and shear thinning, of single agarose and of agarose–alginate hydrogels with those of Pluronic hydrogels, commonly used in bioinks design, to assess their suitability for extrusion bioprinting of cartilage constructs. Even though the single agarose hydrogels (2, 3 and 4% (*w*/*v*)) showed appropriate shear thinning behavior, filament shape fidelity was not the best when compared with 30% Pluronic hydrogels. However, the combination of agarose with alginate in a 3:2 ratio improved print fidelity and demonstrated excellent cell viability for auricular cartilage cells, which was maintained over a 28-day culture period post-bioprinting (>70% cell viability at the end of 28 days) [75]. More recently, Butler et al. [79] explored the combination of agarose with *N*,*O*-carboxymethyl chitosan (NOCC) in different ratios (80:20, 60:40, 40:60, and 20:80) for the development of bioinks laden with neuron cells (neuro2A) (Figure 2C). The rheological properties and printability by extrusion of these bioinks were mainly influenced by the NOCC content, with the agarose-NOCC 40:60 and agarose-NOCC 20:80 bioink formulations presenting the highest storage modulus (20–25 Pa) and printability, evaluated by the assessment of the printability number. However, comparing these two formulations, the post-bioprinting cell viability of neuro2A for 14 days was higher (100%) for the scaffolds produced with the bioink with the highest content of agarose (agarose-NOCC 40:60), which the authors considered to be the best blend in terms of a compromise between mechanical performance and cell viability [79]. This study opens the possibility for future developments in the field of agarose-based bioinks by exploiting the combination of agarose with bioactive compounds or polymers [94,95], as extensively explored for alginate-based bioinks and previously highlighted in Section 2.1.1.

Alternative applications of agarose hydrogels in bioprinting processes, rather than bioink components, have also been explored [93]. At low concentrations (less than 1% (*w*/*v*)), agarose hydrogels can be easily molded and used to cast sub-millimetric geometries [93]. Taking advantage of this feature, Aydin et al. [78] developed a bioink composed of agarose and alginate, produced by a microwave-assisted method, where agarose served as a self-eroding sacrificial part to cast tubular structures within the cell-laden alginate, generating living printed constructs with a vascularized network. Specifically, the use of this sacrificial bioink allowed the bioprinting of a 2 cm tubular structure in only 2 min, which retained shape fidelity and allowed very high cell viability (up to 95%) in a 3-day culture of human adipose tissue-derived mesenchymal stem cells (AT MSCs) [78].

Another use of agarose gels in the bioprinting field is related to their role as a support medium in suspended layer bioprinting, providing protection to fragile and/or complex printed structures from collapsing prior to final crosslinking [76,77]. In this sense, Senior et al. [76] studied the optimization of agarose and gelatin gels for suspended layer 3D printing and bioprinting techniques, with agarose slurry showing more adequate properties, based on rheological studies, for embedded printing of 3D structures than gelatin [76]. In a different study, Cidonio et al. [77] used a 0.5% (*w*/*v*) agarose gel loaded with endothelial growth factor as a support medium for the bioprinting of a laponite-gellan gum bioink for skeletal tissue engineering [77].

### 2.2. Other Plants Derived Polysaccharides

Higher plants are considered one of the main sources of polysaccharides [96]. They produce these natural polymers as structural components of cell walls, e.g., cellulose and pectins, or as a source of energy, stored in the chloroplasts of plant cells, e.g., starch. The high diversity of chemical structures and properties of these plant polysaccharides opens the possibility for their application in several fields, including in the food area (e.g., as gelling agents) [97], in the biomedical field (e.g., in drug delivery systems) [98], and, more recently, in tissue engineering [99]. The interest in using some of these plant-derived polysaccharides in the formulation of bioinks for 3D bioprinting applications has also grown considerably in the later years, as summed up in Table 2, with several works using cellulose and nanocelluloses [100,101,102,103,104,105,106,107,108,109,110,111,112,113,114,115,116,117,118] and pectin [119,120].

#### 2.2.1. Cellulose

**Figure 3:**
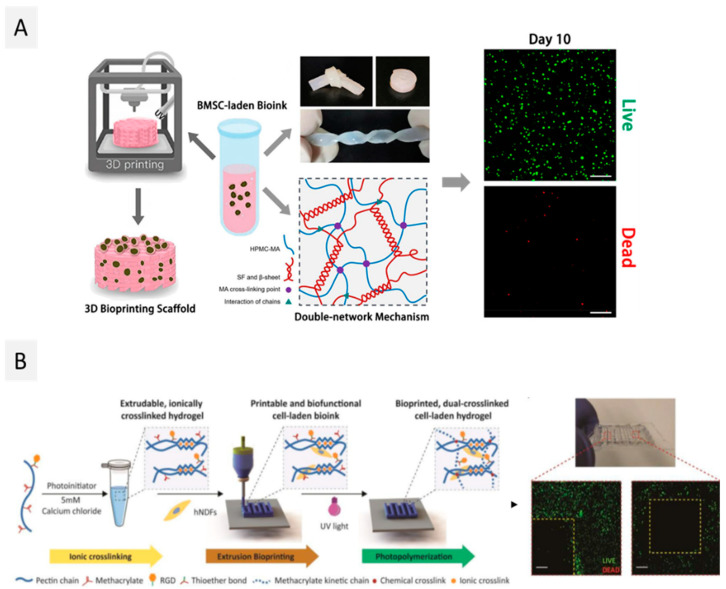
Various approaches for the development of bioinks from higher plant-derived polysaccharides. (**A**) Bioink from hydroxypropyl methylcellulose and silk fibroin for the bioprinting of BMSCs cells (Reproduced with permission from [114]. Copyright American Chemical Society, 2020); and (**B**) bioink of methacrylated pectin with RGD for 3D bioprinting of hNDFs cells (Reproduced with permission from [119]. Copyright Royal Society of Chemistry, 2018).

Cellulose, the major component of plant cell walls, is the most abundant polysaccharide on Earth and consists of β-D-glucopyranose units linked by β-(1,4) glycosidic bonds [121,122], with a variable degree of polymerization (10,000 for native cellulose, 15,000 for cotton plant-fiber cellulose, and after extraction for other plant fibers the range is about 800–10,000, depending on the applied treatment). The high density of free hydroxyl groups contributes to the abundant intra- and intermolecular hydrogen bonds within and between individual chains, promoting their association into cellulose fibers. These cellulose fibers consist of highly ordered (crystalline) and disordered (amorphous) motifs arranged in an alternating fashion. Cellulose fibers are essentially used to produce paper or textiles and, more recently, in the composite industry [123]. However, this strong network of hydrogen bonds is also responsible for the insolubility of cellulose in most common solvents [122]. To overcome this issue, chemical modification of cellulose permits the production of cellulose derivatives with a plethora of properties and applications [124,125]. Carboxymethyl cellulose (CMC), methyl cellulose, ethyl cellulose, hydroxyethyl cellulose, hydroxypropyl methyl cellulose, and mixed ethers such as hydroxyethylmethylcellulose, obtained by reaction of cellulose fibers with alkyl halides in alkaline medium, are some examples of commercially available cellulose derivatives. These cellulose derivatives can be obtained with different degrees of substitution (DS) and, therefore, with a panoply of properties. The use of cellulose derivatives for the development of bioinks has been recently reviewed [126,127], and it is mainly focused on the exploitation of CMC. However, other cellulose derivatives, such as methyl cellulose [101], hydroxyethylcellulose [112], and hydroxypropyl methyl cellulose [113,114], are also starting to be studied in this context. For instance, Ni et al. [114] mixed silk fibroin with hydroxypropyl methyl cellulose, that was previously methacrylated, in different proportions (3:1, 2:2 and 1:3) to bioprint, through extrusion, bone marrow-derived mesenchymal stromal cells (BMSC)-laden double network hydrogels for cartilage tissue repair, as seen in Figure 3A. Regardless of the proportions used, the mechanical properties of the double network hydrogels were improved when compared to the hydrogels contain only silk fibroin or methacrylated hydroxypropyl methyl cellulose. Nonetheless, increasing the silk fibroin (proportion 3:1) resulted in higher mechanical strength, with compressive stress at 50% strain above 100 kPa. The proliferation of BMSCs was addressed using the WST-1 assay, and it was shown that cells proliferated slowly between day 1 and day 7 (0.29 and 0.40, respectively) and then started to proliferate quickly at day 10 (0.86). The LIVE/DEAD assay revealed that the dead cells were up to 46% due to the shear stress imposed on the cells and the UV irradiation at day 1, and decreased to 3% at day 10, which indicates that the formulated hydrogel offers an excellent microenvironment for BMSCs to grow and proliferate.

Additionally, the development of nanocellulose forms, viz. nanofibrillated cellulose (NFC) [128], obtained by the disintegration of cellulose through the combination of intense mechanical treatments and chemical or enzymatic treatments; cellulose nanocrystals (CNC) [129], prepared by acid hydrolysis of cellulose fibers; and bacterial cellulose (BC) produced by non-pathogenic aerobic bacteria [122] also opened the possibility to extend the applications of cellulose to other fields [130]. High surface area, excellent mechanical properties, biocompatibility, and biodegradability are some of the most important features of these cellulose nanoforms, that confer the possibility of using nanocelluloses in biomedical fields, including in 3D bioprinting applications [130]. The use of nanocellulose forms in 3D bioprinting applications has been reviewed [40,46,47], with the more recent papers focusing on different crosslinking strategies to integrate multicomponent nanocellulose-based bioinks [32], the advantages and disadvantages, applications of cellulosic bioinks in printing vascular tissue, bone, and cartilage [49], and other biomedical applications (e.g., drug delivery and wound dressings) [41]. Most of the studies regarding nanocellulose-based bioinks have been focused on the combination of alginate with nanofibrillated cellulose (NFC) in bioink formulations [131], which has already been translated into a commercial bioink, viz. CELLINK Bioink from Cellink [132]; throughout the years, not much evolution and creativity has been observed regarding the use of NFC as a bioink component. Most recent studies still describe its combination with alginate for bioprinting of different cell lines, namely human cartilage [115,116], human dermis [117], bone [102,118], and skeletal muscle [103]. Additionally, nanocelluloses are still mainly used as reinforcing components for hydrogel bioinks due to their excellent mechanical properties. As an illustrative example, Im et al. [102] recently formulated a hydrogel-based bioink using alginate, 2,2,6,6-tetramethylpiperidine-1-oxyl radical (TEMPO)-oxidized cellulose nanofibrils, and polydopamine nanoparticles (with a total solid content of 3% (*w*/*v*)) to produce a functional bioink for MC3T3-E1 cell line printing and bone tissue formation. As expected, the formulated hydrogels showed a shear-thinning behavior. Moreover, the storage modulus was higher than the loss modulus in the range of the tested frequencies (0.1–100 Hz) for all samples, indicating a gel-like behavior. Increasing cellulose nanofibrils content from 0.9 to 2.1% (*w*/*v*) led to an increase in the storage modulus from 541 to 1214 Pa, while the incorporation of 0.5 wt.% polydopamine nanoparticles further increased the storage moduli up to 2.3-fold. Furthermore, cell viability (LIVE/DEAD assay) of osteoblasts was above 80% on day 1 and 7 for all tested bioinks, demonstrating the cytocompatibility of the formulated bioinks.

The exploitation of cellulose nanocrystals (CNCs) has been described more recently, namely through their combination with alginate [133] or other biopolymers/compounds (platelet lysate [104], gelatin methacrylate/hyaluronic acid methacrylate [110], carrageenan [106], and chitosan [105]). For example, Boonlai et al. [106] blended CNCs, *k*-carrageenan, and methylcellulose, and a suitable hydrogel was created through ionic crosslinking (with aqueous KCl) for 3D extrusion-based bioprinting of living constructs for bone tissue engineering and regeneration applications. The addition of 2 and 4 wt.% of CNCs into the hydrogels led to better shear-thinning behavior since nanocellulose improved the rheological properties of the bioink. Moreover, CNCs-reinforced hydrogels showed an increase in compressive stress at 30% strain from 20.03 ± 0.02 to 23.28 ± 0.01 kPa when increasing CNCs content from 2 to 4 wt.%, respectively. Five days after bioprinting, the viability of L929-laden printed constructs was higher than 90%, indicating that both formulated biomaterials and the bioprinting process were not harmful to these cells. Another interesting work by Maturavongsadit et al. [105] explores the use of these cellulose nanoforms for reinforcement of a chitosan-based bioink, which will be detailed in Section 2.4.

Bacterial cellulose (BC), although obtained from microbial sources, will be addressed in this section, given its relevance as a nanocellulosic reinforcement agent. This is the least explored nanocellulose form in 3D bioprinting, with only two reported studies [107,108]. This could be due to the fact that, in order to be used for the development of bioinks, BC needs to be disintegrated to form a suspension [32]. For instance, Wu et al. [107] used extrusion-based 3D bioprinting technologies to fabricate a nerve scaffold composed of neuronal Schwann (RSC96) cells laden in a sodium alginate-GelMA-BC-based hydrogel. A thixotropic evaluation revealed that, at 0.1 s^−1^, the ink with BC had a viscosity of 4 × 10^6^ Pa⋅s, which was higher than the formulations without BC. Increasing shear rate led to an accentuated decrease in viscosity, which was rapidly recovered after the shear rate was restored to 0.1 s^−1^. Furthermore, the incorporation of BC in the hydrogels improved the mechanical properties, with compression modulus increasing from 2.25 kPa for GelMA to 10.92 kPa for GelMA-BC-based hydrogels. LIVE/DEAD assay revealed high cell viability since most of the cells seen in fluorescence microscopy were stained in green, and from day 1 to day 7, the number of fluorescent cells increased. However, no quantification of viable cells was carried out. Cells were able to grow and form linear connections, a phenomenon more evident in BC-based hydrogels, suggesting that BC promoted the oriented growth of RSC96 cells and the adhesion to sodium alginate-GelMA hydrogel.

#### 2.2.2. Pectin

Pectin is an anionic polysaccharide present in fruits, such as apple pomace and citrus peel, and in vegetables, and it is composed of three polysaccharide domains, viz. homogalacturonan, rhamnogalacturonan-I, and rhamnogalacturonan-II [134,135]. Homogalacturonan is the major domain and contains α-(1,4)-D-linked galacturonic acid units, with differing degrees of methylation of the uronic acid residues and molecular weights between 50 and 150 kDa. Pectins with low methylation degrees form hydrogels in the presence of multivalent ions (e.g., Ca^2+^, Mg^2+^, Fe^3+^, Cu^4+^), whereas pectins with higher methylation degrees form hydrogels by the establishment of hydrogen bonds and hydrophobic interactions at low pH and with the addition of different sugars (e.g., sucrose or glucose) [136,137]. Pectin hydrogels are non-toxic and have been used in various fields of biomedicine, including bandages, soft contact lenses, and drug delivery systems, as recently reviewed by Li et al. [138]. The role of pectin as a promising polysaccharide for the development of bioinks for 3D bioprinting has also been mentioned by Jovic et al. [139] who reviewed plant-based biomaterials for 3D bioprinting and other biomedical applications, and by Indurkar et al. [140], who summarized plant-based biomaterials for tissue engineering.

Even so, the applications of pectin in 3D bioprinting only started to be explored in 2018 by Pereira et al. [119]. In this study, pectin was methacrylated (PECMA) to allow the binding of integrin motifs (by biofunctionalization with a cell-adhesive peptide containing the amino acid sequence RGD) and the formation of hydrogels by UV photopolymerization (Figure 3B). Increasing PECMA concentration from 1.5 to 2.5 wt.% led to an increase in G’ values from 0.0769 ± 0.0077 kPa to 2.6 ± 0.3 kPa, respectively. Furthermore, PECMA (1.5 wt.%) formulations were incubated with different concentrations of CaCl_2_ (0–5 mM), and ionic pre-crosslinking resulted in a significant increase of the yield stress from 1.18 Pa at 3 mM and to 9.16 Pa at 5 mM, with the concentration allowing the ink to form a continuous filament when printing by extrusion. The final UV crosslinking after printing increased the stability of the obtained constructs. The cell viability of human neonatal dermal fibroblasts (hNDFs), laden on this PECMA bioink, was qualitatively evaluated by analysis of the LIVE/DEAD assay, and the results showed that after 24 h post-printing, the printed constructs displayed viable cells. In this study, quantification was also not considered, and higher post-printing periods were also not considered for this characterization. More recently, Hu et al. [120] explored the incorporation of pure pectin in alginate-Pluronic-based hydrogels to reduce the inflammatory response to cell replacement therapies and later implanted it in mice. The addition of pectin did not influence the viscoelastic properties of the formulated hydrogel bioinks since they had similar elastic modulus (stiffness) as the ones without pectin (around 190 kPa). Additionally, pectin was able to protect the bioprinted insulin-producing insulinoma (MIN6) cells from inflammatory stress, which was evidenced by the higher cell viability (73.3 ± 3.7%) when compared with pectin-free constructs (64.4 ± 1.8%). This work highlights the role of pectin in reducing inflammatory responses, which can be further explored in the future in other bioink formulations.

#### 2.2.3. Starch

Starch is a neutral polysaccharide produced by plants, such as rice, wheat, and maize, in the form of insoluble granules that constitute their main energy source [141]. In disregarding the plant source, starch is composed of two different polysaccharides: amylose (linear chain composed of (1,4)-linked α-d-glucan, with a molecular weight of 10^5^ g·mol^−1^) and amylopectin (branched α-d-glucan, with a molecular weight of 10^6^–10^7^ g·mol−1). At room temperature, this polysaccharide is insoluble in water and forms a suspension [142]. Nonetheless, upon heating, the granules swell and gelatinize. This promotes the separation of the amylose fraction from amylopectin and the emergence of a continuous phase surrounding the swollen granules. By cooling the starch suspension, the amylose phase separates, leading to gel formation. The resulting gel is highly stable and biocompatible, and, therefore, it has been used in the biomedical field, viz. in drug delivery systems [143]. However, to the best of our knowledge, starch has never been used in the development of cell-laden bioinks for 3D bioprinting applications. One reason could be related to the fact that heat treatments (100 °C) are often needed to dry and maintain the integrity of starch printed structures, as highlighted by Aljohani et al. [144] and Carrow et al. [145].

Recently, Maniglia and co-workers [146] explored the modification of cassava starch by using an ozone process to evaluate the potential of starch to produce hydrogels for 3D food printing. Ozonation time originated a modified starch with higher carbonyl and carboxyl contents, acidic character, and reduced molecular size, leading to hydrogels with different behaviors depending on the extent of the ozonation but adequate for extrusion 3D printing. Following a different strategy, Noè et al. [147] used starch methacrylate to generate a photocrosslinkable hydrogel and evaluated its processability by photocuring in a mold or by digital light processing (DLP) 3D printing. This modification strategy allowed the authors to obtain hydrogels with good mechanical and rheological properties and to print starch structures without the need for any additional heat treatment. In a different vein, pure starch was combined with gellan gum to formulate 3D-printed scaffolds with various printing gaps for seeding Schwann cells [148]. Results indicated that the printed constructs were stable, with adequate swelling ratios, and were non-cytotoxic toward the L929 fibroblast cell line. These approaches will certainly potentiate the future use of starch in the formulation of cell-laden bioinks for 3D bioprinting applications, due to their suitability for 3D bioprinting as, in fact, explored for other polysaccharides.

### 2.3. Microbial Derived Polysaccharides

Several microorganisms produce polysaccharides that act as storage components or participate in distinct biological processes, such as cell adhesion, molecular recognition, and cell–cell interaction [149]. Microbial polysaccharides can accumulate inside the microbial cells, the so-called intracellular polysaccharides, such as glycogen [150], or be secreted to the surrounding or synthesized extracellularly, and these are known as exopolysaccharides or extracellular polysaccharides (e.g., dextran, xanthan gum, gellan gum, pullulan, and bacterial cellulose) [149]. Here, only exopolysaccharides (except for bacterial cellulose that was considered in Section 2.2) will be reviewed since they are the most commonly used microbial polysaccharides for bioprinting applications [26]. The usage of these polysaccharides in 3D bioprinting is still gaining ground since the exploitation of microbial-derived polysaccharides-based bioinks has only been recently described in the literature [151,152,153,154,155,156,157,158,159,160,161,162], as summarized in Table 3.

#### 2.3.1. Dextran

Dextran is an exopolysaccharide produced by several bacteria, such as *Leuconostoc meenteroides*, *Lactobacillus brevis*, and *Streptococcus mutans*, and its secretion is used by bacteria to form biofilms or protective microbial coatings [163]. Dextran is a branched polysaccharide composed of glucose units linked consecutively by α-(1,6) linkages, and α-(1,3), and occasionally α-(1,4) or α-(1,2) branched linkages, with a molecular weight ranging between 1 and 40,000 kDa [164]. However, since pure dextran cannot form hydrogels, it must be chemically modified, either by functionalization with methacrylate groups or by oxidation techniques [165], to enable crosslinking.

**Figure 4:**
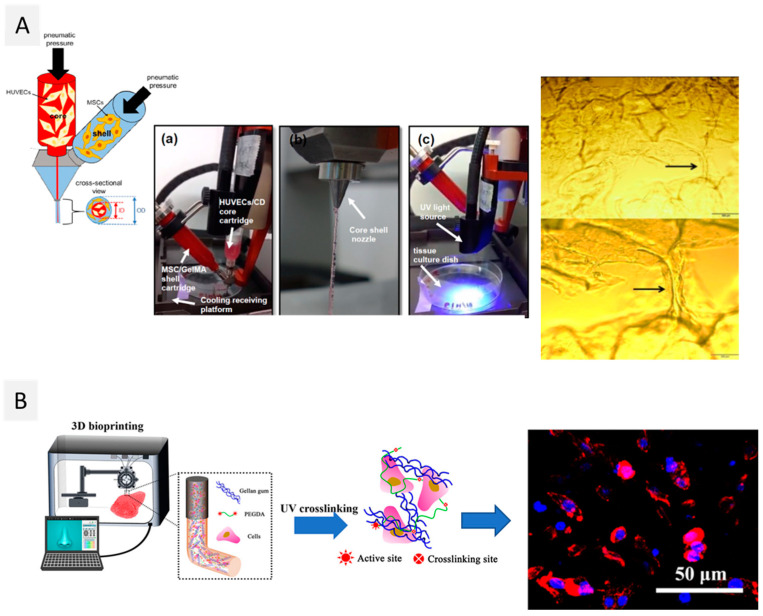
Diverse strategies for the creation of novel bioinks from microbial derived polysaccharides. (**A**) Core/shell bioinks composed of dextran aldehyde and succinylated chitosan (core), and GelMA (shell) for the bioprinting of MSCs and HUVECs cells (Reproduced with permission from [151]. Copyright American Chemical Society, 2020); (**B**) UV-crosslinked bioinks gellan gum and PEGDA with BMSCs and MC3T3-E1 cells (Reproduced with permission from [159]. Copyright Elsevier, 2018).

In fact, to date, only one study reported the use of oxidized dextran for the bioprinting of a vascularized construct for wound care, using a promising core/shell extrusion-based 3D bioprinting technology [151]. To achieve that, peptide-functionalized succinylated chitosan (C) and periodate oxidized dextran (D)-based hydrogel was used as the core, and GelMA was used as the shell (Figure 4A). Two types of cells were used in this work: HUVECs in the core and BMSC in the shell. The 3D bioprinted peptide-CD/GelMA constructs provided an appropriate microenvironment for cell growth and differentiation, with HUVEC-specific markers detectable 21 days after bioprinting, demonstrating the presence of endothelial cells within the tube-like structures. Furthermore, osteogenic differentiation indicated that the components of the constructs did not affect BMSC multi-potency, which is important to create regenerative constructs that may differentiate into another cell type as needed. Despite being the sole study to date on the use of dextran-based bioinks, this work opens the door to the use of this polysaccharide to produce vascularized structures, which is still one of the main challenges in the 3D bioprinting of living tissues [85].

#### 2.3.2. Xanthan Gum

Xanthan gum is a negatively charged exopolysaccharide with an average molecular weight close to 2000 kDa, synthesized by *Xanthomonas campestris* bacteria, composed of a 1,4 linked β-D-glucose main chain, with trisaccharide side chains composed of β-D-mannose, β-D-glucuronic acid, and α-D-mannose [166]. In aqueous solutions, xanthan gum presents two different conformations depending on the temperature: an ordered and rigid double helical strand structure with a gel-like behavior at low temperature (below 40–50 °C) and a disordered and flexible coil structure at higher temperatures (above 50 °C). A drawback of xanthan gum is the formation of aggregates in water, upon dispersion, due to inadequate hydration [167]. As a result, a gelatinous outer layer is formed, which blocks the infiltration of water and, therefore, compromises the complete dissolution of this polysaccharide. To overcome this issue, xanthan gum may be modified or combined with other polymers (through crosslinking processes) to improve its water solubilization [168]. Despite this issue, xanthan gum is frequently employed as a viscosity regulator due to its excellent rheological characteristics. As a result, it constitutes an excellent option to improve the rheological and mechanical characteristics of bioinks. However, its application in the fabrication of bioinks for 3D bioprinting is still relatively new, with just three published papers on the topic [154,155,169].

Specifically, Lim et al. [152] benefited from the shear-thinning properties of xanthan gum and combined it with CMC in different concentration ratios (1:1, 1:2, 2:1, and 2:2) and with alginate, GelMA, and hMSCs for the development of an adequate bioink for extrusion-based bioprinting. The viscosity of the bioinks decreased as the shear rate increased, confirming the contribution of xanthan gum to the shear-thinning behavior. Furthermore, all formulations showed a gel-like behavior as the G’ was higher than the loss modulus (G”) within the tested region (1–100 rad/s). Two different crosslinking methods were evaluated, namely UV irradiation and UV plus ionic crosslinking (with Ca^2+^). No differences were observed in the viabilities of the hMSCS cells on the bioprinted constructs crosslinked with the two distinct approaches (with cell viability >80%). However, considerably higher cell proliferation was detected for the hydrogel constructs crosslinked under the UV and ionic conditions, as evaluated using the bromodeoxyuridine (BrdU) assay. These results were attributed by the authors to differences in pore size and distribution, which may affect cell infiltration behavior and proliferation. The combination of xanthan gum with GelMA is also the base of two commercial bioinks, namely GelXA and GelXG, commercialized by Cellink company [169,170].

In another study, Muthusamy et al. [155] also explored the thickening properties of xanthan gum, conjugating this polymer with neutralized collagen type 1 to develop a bioink for bioprinting endothelial cells in specific spatial locations, sandwiched between bioprinted layers of fibroblasts to promote vessel formation. The rheological evaluation showed that the addition of xanthan gum (0.5–10% (*w*/*v*)) contributed to a higher viscosity (up to 1088.8 mPa·s for the formulation with 10% of xanthan gum) and a higher G’, enhancing the printability of the collagen-based formulations. The bioink showed a shear-thinning and a gel-like behavior, as G’ values were higher than G”. Furthermore, cell-laden bioprinted constructs displayed overall high cell viability (92.39% ± 2.02% at 24 h post printing and 89.40% ± 2.58% at 48 h post-printing). Endothelial sprouting and formation of interconnected capillary-like networks within the lattice were also observed by day 6. More recently, Piola et al. [154] prepared xanthan gum/gelatin-based bioinks, with different proportions of the biopolymers, laden with human keratinocytes and fibroblasts to produce scaffolds for cell growth or wound dressings [154]. Here, glutaraldehyde was used to crosslink gelatin. As expected, higher amounts of gelatin (2.5 and 3% (*w*/*v*)) and xanthan gum (1.2% (*w*/*v*)) contributed to higher shape retention of the bioprinted structures. However, the glutaraldehyde crosslinking of gelatin was essential to maintain the shape of the constructs. The bioprinting process of the cell-laden bioinks did not affect the cell viability, as no sign of cell death was visible on day 1 after bioprinting. In fact, cell proliferation increased until 14 days of culture [154].

#### 2.3.3. Gellan Gum

Gellan gum is an anionic extracellular bacterial polysaccharide produced by microbial fermentation of *Sphingomonas paucimobilis*, composed of a repeating unit of β-(1,3)-D-glucose, β-(1,4)-D-glucuronic acid, β-(1,4)-D-glucose, and α-(1,4)-L-rhamnose, with a molecular weight of 500 kDa [171]. This polysaccharide is also thermo-responsive, as it exists in coil form in solutions at temperatures above 60 °C [172]. Below this temperature, it evolves into a double-helix form, producing a hydrogel. Moreover, the presence of carboxylic groups in its structure allows the formation of hydrogels in the presence of mono and divalent ions, such as Na^+^, K^+^ and Mg^2+^, Ca^2+^, respectively [173]. Gellan gum hydrogels obtained by ionotropic crosslinking are brittle and mechanically weak, so it is normally necessary to chemically modify [157] or blend them with other polymers [153,158,159,160,161,162] to obtain printable bioinks.

The shear-thinning properties of gellan gum were explored for the first time in 2014 [156] for the biofabrication of living tissue constructs by extrusion-based 3D bioprinting of MSC-laden polylactic acid microcarriers encapsulated in gelatin methacrylate-gellan gum (GelMA-GG) hydrogel bioinks. As GelMA is not sufficiently viscous, gellan gum was added to increase the solution viscosity and improve the printability of the bioink. Actually, the inclusion of gellan gum helped to keep the form of the printed filaments after they were deposited, permitting the fabrication of structures with high shape fidelity. Furthermore, the viability of MSC cells 3 days after bioprinting was higher than 90%, and cells were homogeneously distributed within the hydrogel matrix. More recently, Wu et al. [159] combined the shear-thinning and recovery properties of gellan gum with the rapid photocrosslinking of poly(ethylene glycol) diacrylate (PEGDA) to develop a double network hydrogel to produce human-scale constructs (viz. a human ear and nose) with high-fidelity (Figure 4B). All formulations presented shear-thinning performance and a gel-like behavior, with G’ > G” under small strain and a liquid-like behavior (G” > G’) under large strain. These hydrogels were laden with murine BMSCs and MC3T3-E1 cells. During the 21 days of cell culture, both cell types remained stable and viable (cell viability above 87%). Plus, the bioprinted scaffold provided an open network with sufficient exchange of oxygen and nutrients, promoting cell activity, measured by integrated optical density (IOD). Similarly, Zhuang et al. [160] combined GelMA with gellan gum as a viscosity enhancer to improve the printability of the inks and shape fidelity of the correspondent constructs. However, GelMA-GG printability is still narrowed to single and thin structures. As an improvement, Zhuang and co-workers introduced a new printing strategy that allows the fabrication of more complex structures via UV-assisted extrusion printing technology. Printability analysis allowed the selection of six combinations of GelMA-GG inks (5–0.5%, 7.5–0.1%, 7.5–0.2%, 7.5–0.5%, 10–0.1%, and 10–0.2% (*w*/*v*)) that showed good printability, good cell encapsulation, and negligible cell sedimentation. As expected, higher polymer concentration led to a higher compressive modulus, as was seen by the increment of the compressive modulus from 9 to 16 kPa for this set of formulations. Bioprinting C2C12 cells in 5–0.5% (*w*/*v*) GelMA-GG led to a faster proliferation rate when compared to the cells bioprinted in 7.5–0.5% (*w*/*v*) GelMA-GG, as indicated by the increased cell number after 7 days (>160,000 cells), possibly due to more favorable micro-structure and stiffness of the materials.

Methacrylated derivatives of gellan gum can also be prepared. In fact, a commercialized bioink is based on GGMA (GumMA from AdBioInk) [174].

#### 2.3.4. Pullulan

Pullulan is a non-ionic exopolysaccharide, mainly produced by fermentation of *Aureobasidium pullulans*, consisting of maltotriose units (3 glucose units linked through α-(1,4) glycosidic bonds) linked through α-(1,6) bonds, with a molecular weight between 10 and 400 kDa [175,176]. Pullulan can be synthesized as well as by other microorganisms such as *Tremella mesenterica* and *Cryphonectria parasitica*. Pullulan is a highly water-soluble polysaccharide that originates low viscosity solutions. Pullulan could also be chemically modified, for instance, giving origin to methacrylate derivatives, to produce hydrogels. As an example, Qin et al. [177] functionalized pullulan with methacrylate moieties and combined it with poly(ethylene glycol) diacrylate (PEGDA) to form a hydrogel for cartilage tissue engineering. Similarly, Giustina et al. [178] also combined pullulan methacrylate with PEGDA for multiscale light-assisted 3D printing techniques, such as stereolithography and two-photon lithography. Additionally, pullulan was functionalized with fibronectin, a high molecular weight glycoprotein, to originate active sites for cell attachment. Cell viability of MSCs was assessed, and the addition of fibronectin allowed the attachment of mesenchymal stem cells and contributed to cell viabilities above 70% for different timeframes after cell seeding (24, 48, 72, and 168 h). Similarly, Mugnaini et al. [177] also described the synthesis of a photocrosslinkable pullulan derivative through the introduction of methacrylic groups for exploitation in 3D printing. The modification of pullulan did not affect the rheologic properties of the polysaccharide, especially the shear-thinning behavior, granting this polysaccharide the ability to be printed into self-standing printouts. Even though these studies evidence the potentialities of pullulan to produce hydrogels formulations with suitable properties for extrusion 3D printing, to the best of our knowledge, the use of pullulan for the preparation of cell-laden bioinks for 3D bioprinting has never been reported.

### 2.4. Crustacean Derived Polysaccharides

The use of renewable and sustainable marine resources for 3D bioprinting endeavors is not restricted to seaweed-derived polysaccharides, as described above in Section 2.1. In fact, the shell of crustaceans, such as shrimps and crabs, is a very important source of chitin, which is often considered the second most abundant polymer after cellulose [179]. Chitin is a polysaccharide composed of β-(1,4)-linked *N*-acetyl-D-glucosamine residues, commonly present on the exoskeletons of crustaceans but also in insects and in the cell walls of fungi [180]. Chitin is biodegradable, nontoxic, and possesses renowned antibacterial properties that grant this polysaccharide numerous applications in the biomedical field, including in drug delivery [181] and tissue engineering approaches [182].

However, only the work of Li et al. [183] seems to have described, to this date, a chitin-based bioink for 3D bioprinting. The limited use of chitin in this domain is certainly related to its lower solubility in most common solvents, implying challenging processability issues. In fact, this study involves the use of a chitin derivative, viz. hydroxypropyl chitin. Specifically, hydroxypropyl chitin was used together with Matrigel (a gelatinous protein mixture secreted by Engelbreth-Holm-Swarm mouse sarcoma cells [5]) to form a hydrogel bioink for the 3D bioprinting of constructs containing induced pluripotent cells (iPSCs). The addition of Matrigel was justified by the authors since hydroxypropyl chitin has no RGD groups in its composition to promote cell adhesion. The thermos-sensitivity of this bioink was proven by the continuous increase in storage and loss moduli with increasing temperature, reaching a maximum storage modulus of 432 Pa, 654 Pa, and 958 Pa for 2%, 2.5%, and 3% (*w*/*v*) of hydroxypropyl chitin, respectively. Additionally, it was observed that the bioinks containing a higher concentration of hydroxypropyl chitin contributed to a smaller variance in cell aggregate size, as obtained for the bioink with 3% (*w*/*v*) of this polysaccharide (124.1 ± 28.2 µm). All bioinks revealed high cell survival after bioprinting (>90%), and those with higher Matrigel content showed good cell proliferation after 10 days (at around 30%) [183].

However, chitosan is the most remarkable derivative of chitin, obtained through the enzymatic or chemical deacetylation of the starting material to variable extents (higher than 50%). Typically, chitosan is obtained with a molecular weight of 50 to 2000 kDa, and because of the deacetylation, it is positively charged and soluble in slightly acidic aqueous solutions, and it is well-known for its antimicrobial properties [179,184]. Chitosan forms hydrogels by treatment with multivalent anions, such as glycerophosphate and tripolyphosphate [185]. It is also biodegradable and biocompatible, with low immunogenicity, and because of all these features, it has been widely studied for many biomedical applications, such as drug delivery [186] and wound healing [187,188]. The development of bioinks for 3D bioprinting is no exception, as very recently reviewed by Taghizadeh et al. [44] in a work that thoroughly explores this topic, highlighting the reasons for the use of chitosan-based bioinks, the challenges and limitations of these materials, and their applications in the biomedical field. Chitosan-based bioinks have already been explored to fabricate 3D living structures laden with a panoply of cells for use in neural networks, bone, and skin tissue regeneration, among others [44]. Moreover, the emergence of commercially available chitosan-based bioinks (viz. Chitoink by Cellink [189]) is a clear sign of the growing interest in this polysaccharide.

The use of chitosan in bioink formulations explores its unique features, namely the biological properties (antimicrobial properties) and the ease of functionalization (to introduce different reactive moieties, i.e., allowing different crosslinking strategies). In Table 4, illustrative works on the use of chitosan for the development of new bioinks are summarized [190,191,192,193,194,195,196,197].

**Figure 5:**
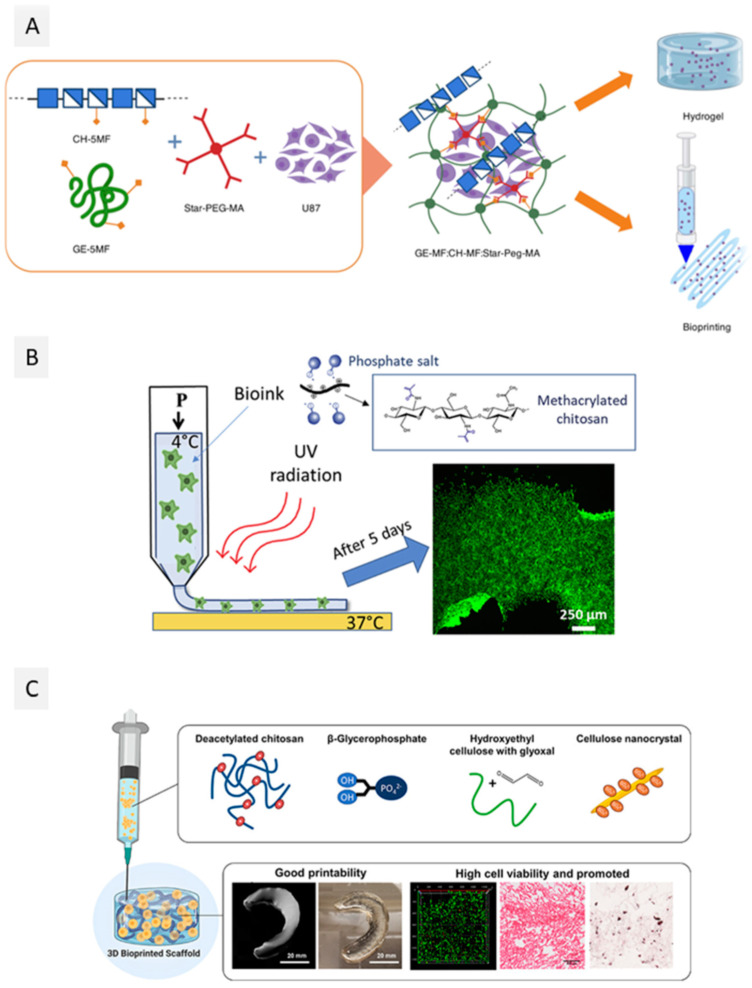
Different approaches for the development of chitosan-based bioinks. (**A**) Example of the modification of chitosan (and gelatin) with 5-methyl furfural and crosslinking with Star-PEG-MA for the development of a bioink for 3D bioprinting with U87 cells (Reproduced with permission from [194]. Copyright Frontiers, 2020); (**B**) dual-crosslinked hydrogel bioink using chitosan methacrylate together with β-glycerol phosphate with NIH 3T3 cells (Reproduced with permission from [197]. Copyright Elsevier, 2020); and (**C**) bioink based on chitosan, together with β-glycerol phosphate and hydroxyethyl cellulose, reinforced with cellulose nanocrystals, with good printability and high cell viability(Reproduced with permission from [105]. Copyright American Chemical Society, 2021).

Magli et al. [194] functionalized chitosan and gelatin with 5-methyl furfural, aiming to produce hydrogels through crosslinking via Diels-Alder thermoreversible reaction with poly(ethylene glycol)-Star-maleimide (Star-PEG-ma) (Figure 5A). In the linear viscoelastic zone, the intrinsic structural characteristics of the hydrogels were independent of the applied stress, with G’ value higher than the G”, and this region was linear up to about 40% of strain. When the strain ceased, the samples exhibited a solid gel response, with G’ values being restored to 90% of the original value. The extrusion-based bioprinting process of the U87 (human primary glioblastoma) cell-laden bioink resulted in a construct that remained stable until 6 days of culture, with good cell viability at day 1 (80%) and enhanced cell viability at days 3 and 6 (>80%), with clear cell proliferation in the hydrogel [194]. Following a different approach, Tonda-Turo et al. [197] developed a chitosan-based bioink with a dual crosslinking mechanism that combines thermally induced gelation and photocrosslinking. For this purpose, chitosan methacrylate and β-glycerol phosphate salt were added to provide thermo-sensitive behavior (Figure 5B). The photocrosslinking was found to yield hydrogels with an elastic modulus of around 6 kPa. Extrusion bioprinting of these hydrogels laden with NIH 3T3 cells originated 3D constructs with good dispersion of the cells, even after 24 h; after 48 h, cell proliferation was observed [197]. However, even though the authors claim that this bioink allows cell proliferation and organization in tissues, no precise evaluation of cell viability post-bioprinting was performed.

Another common strategy to improve the performance of chitosan-based bioinks is the concomitant use of other components or nanostructures in the bioinks. The work of Pisani et al. [196], for instance, describes the development of a bioink based on chitosan together with poly(gamma-glutamic acid) γPGA. The hydrogel was formed by the ionic interaction between the amine groups of chitosan and the carboxylic acid groups of γPGA, resulting in the gelation of a 4.5% (*w*/*v*) chitosan solution just 50 s after the addition of a 2% (*w*/*v*) γPGA solution. The authors used this hydrogel for the 3D bioprinting of grid-shaped constructs with human dermal fibroblasts, and cell viabilities of around 70% were seen 24 h after the bioprinting process, and kept for 14 days in culture [196].

As already described in the section dedicated to cellulose (Section 2.2.1), the use of cellulose nanoforms is a common strategy to reinforce the bioinks developed from other biopolymers. Chitosan is no exception, and the very recent work by Maturavongsadit et al. [105] uses this approach by describing a bioink based on chitosan, with glycerophosphate and hydroxyethyl cellulose, reinforced with CNCs for the 3D extrusion bioprinting of constructs containing MC3T3-E1 cells (Figure 5C). The authors saw an improvement in the rheological properties of the bioinks and the mechanical properties of the constructs with the addition of CNCs, with a 1.5-fold increase in Young’s modulus for the bioinks with a higher concentration of these cellulose nanostructures. On the other hand, cell viabilities were not impacted by the presence of CNCs in the constructs, and, interestingly, the analysis of osteogenic differentiation showed that the bioinks with higher CNCs content (1.5% (*w*/*v*)) showed a faster onset of osteogenesis, higher formation of ECM, and higher calcium deposition at day 21. This increase in osteogenic markers is justified by the authors with the significantly higher mechanical properties of the structures attributed to the presence of CNCs [105].

### 2.5. Glycosaminoglycans

Glycosaminoglycans are polysaccharides composed of repeating units of 2-amino-2-deoxy sugars. They are naturally present in the ECM in their integral and single form or covalently bound to proteins to form proteoglycans. Most glycosaminoglycans are obtained from animal sources (such as rooster combs and shark cartilage), but microbial approaches (including production via fermentation by *Streptococci*) are increasingly explored [198,199].

Since they are the building blocks of the natural ECM, glycosaminoglycans are of extreme interest in producing biomimetic cell culture models and tissue constructs [200]. In fact, hydrogels obtained from these polysaccharides have found a wide array of applications in the biomedical domain [201]. Chondroitin sulfate, dermatan sulfate, heparin and hyaluronic acid are some examples of glycosaminoglycans that have been used as components of hydrogels to encapsulate and culture living cells [201]. However, hyaluronic acid is by far the most studied glycosaminoglycan in this area. Hyaluronic acid is constituted of repeating disaccharide units of D-glucuronic acid and *N*-acetyl- D-glucosamine, with a molecular weight between 100 to 10,000 kDa, depending on the source. It is the main component of the ECM of cartilage, and it is also present in the epithelial, neural, and connective tissues of vertebrates [202]. The several attractive properties of this biopolymer rely on its high viscoelasticity, biodegradability, and low immunogenicity, allied with its unique biological interaction with cells, and they have been widely explored in the biomedical field [202]. As far as bioprinting applications are concerned, hyaluronic acid is also the most explored glycosaminoglycan, as reviewed by Petta et al. [31]. This appraisal highlights the latest advances in hyaluronic acid-based bioinks for extrusion 3D bioprinting divided into four categories: (i) bioinks where hyaluronic acid or its derivatives are the main component and self-standing material; (ii) blends of hyaluronic acid derivatives with both natural polymers or synthetic polymers; (iii) incorporation of hyaluronic acid or its derivatives in ink formulations to improve both the viscosity, the final mechanical stability or the biological properties; or (iv) blends with mechanically competent support materials.

**Figure 6:**
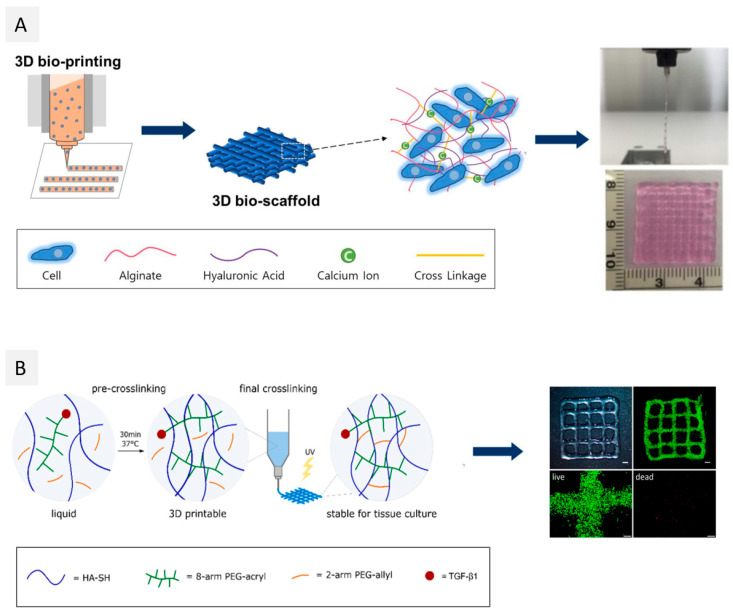
Some strategies applied for the development of hyaluronic acid-based bioinks. (**A**) Bioink of hyaluronic acid and alginate for the bioprinting of NIH 3T3 cells. (Reproduced with permission from [203]. Copyright MDPI, 2021); and (**B**) bioink based on thiol-modified hyaluronic acid and a growth factor (TGF-β1) for the bioprinting of MSCs for chondrogenic differentiation (Reproduced with permission from [205,206]. Copyright John Wiley & Sons, Inc. and MDPI, 2022).

Although most of the previous works on this topic are reviewed in that recent appraisal, some more recent studies [203,204,205,206] published lately are worthy of note, and they are described below. The work of Lee et al. [203], for instance, describes the development of bioinks from hyaluronic acid and sodium alginate at different ratios (Figure 6A). The authors saw an increase in the viscosity of the bioinks with increasing hyaluronic acid content (with bioinks S100H0, S90H10, and S70H30 revealing viscosities of 883, 1211, and 1525 Pa·s, respectively) and were able to bioprint 3D-constructs through extrusion techniques using all the formulations. The evaluation of the viability and proliferation of NIH 3T3 cells in the constructs showed living and well-spread cells after 7 days for all bioinks, and the presence of a higher content of hyaluronic acid led to increased proliferation of these cells in the scaffolds after 4 days [203]. Ma et al. [204], on a similar approach, combined hyaluronic acid and alginate with different concentrations of gelatin, aiming to create models of the brain matrix microenvironment. The authors printed the bioinks at 37 °C in order to preserve the viability of human glial cells (HEBs) and found that the bioink C (with 0.015 g/mL HA, 0.015 g/mL alginate and 0.075 g/mL gelatin) originated a construct with close stiffness to that of human brain tissue, and maintained cell viabilities above 85% after 14 days [204]. Both works confirm the approach described above of using hyaluronic acid together with other polymers to improve the bioinks’ features.

Nonetheless, the modification of hyaluronic acid is also a logical strategy for these endeavors, providing this polysaccharide with new features. In fact, Sigma-Aldrich commerciallizes a bioink kit based on a methacrylated derivative of hyaluronic acid (PhotoHA™-IRG) [207]. In this topic, the recent and very interesting works by Hauptstein et al. [205,206] described the development of hyaluronic acid-based bioinks using thiol-modified hyaluronic acid and its crosslinking with acrylated (PEG-diacryl) and allylated (PEG-diallyl) polyethylene glycol in a two-step reaction. The resulting hydrogels were later used together with tethered TGF-β1 growth factor for the bioprinting of cartilaginous tissue constructs (Figure 6B). The encapsulated MSCs resisted the extrusion procedure, with the quantification of cell survival immediately after bioprinting revealing that around 98% of the cells were still alive in the constructs. After culture for 21 days, the authors observed notorious cell survival and enhanced chondrogenic differentiation for the hyaluronic acid-based constructs containing the tethered growth factor [206].

## 3. Conclusions and Future Perspectives

Three-dimensional bioprinting shows great potential for the tailored fabrication of artificial living tissues, with several applications in the biomedical field. This technique has seen much development in recent years, yet one of the main challenges of 3D bioprinting is still the necessity of bioink formulations with appropriate features. Understandably, while designing novel bioinks, special attention is given to their mechanical and rheological properties and cell compatibility. Therefore, hydrogel-based bioinks are one of the most investigated classes, given their similarity to the ECM and their unique structure that provides permeability to oxygen and nutrients and a friendly environment for cells to thrive. Polysaccharides constitute a remarkable family of polymeric raw materials for the creation of new hydrogel-based bioinks due to their intrinsic traits of biodegradability, nontoxicity, tailorable chemistry, and diverse crosslinking mechanisms that are rather appealing for 3D bioprinting applications, as proven by the number of works analyzed in this review. Polysaccharides-based hydrogel bioinks have been used to print a panoply of cells, including Mesenchymal Stem Cells (MSCs), Human Umbilical Vein Endothelial Cells (HUVECs), and preosteoblasts (MC3T3), among many others, envisioning the 3D bioprinting of living tissues for different applications.

Polysaccharides with carboxylic (or carboxylate) and sulfate groups that are easily crosslinked with mono or divalent cations, e.g., alginate, carrageenan, and pectin, are normally used as polymeric matrices of hydrogel bioinks. However, the obtained hydrogels typically lack the desired rheological properties and the printed constructs for mechanical performance and long-term stability. One of the strategies used to overcome this limitation is the production of composite materials, and here, cellulose, the most abundant polysaccharide, and in particular its nanometric forms, have a major role. In this topic, a panoply of other polysaccharide nanometric forms, namely starch nanocrystals and chitin nanocrystals or nanofibers, or even protein nanofibrils, which are gaining considerable attention in other applications, could also be a good choice for new bioink formulations. The combination of these polysaccharides with other biopolymers (e.g., proteins, such as gelatin and collagen or even other polysaccharides) also circumvents the above-mentioned limitations. However, improvements on the biological performance using this strategy have also been explored by several research teams. Polysaccharides with thickening properties, such as, for instance, gellan and xanthan gums, are normally used to improve the rheological properties of other biopolymeric hydrogel bioinks.

Another main challenge in the use of polysaccharide-based hydrogel bioinks is the fact that many of these biopolymers need to be modified to be used for such endeavors, granting the polysaccharides with different or improved functionalities (viz., cell adhesion and proliferation, e.g., by grafting of RGD moieties), e.g., alginate or agarose, crosslinking abilities, e.g., pullulan, starch, and hyaluronic acid, or even improved solubility, e.g., cellulose and chitin. In fact, the chemical modification with polymerizable moieties, mainly acrylate groups, to allow double crosslinking strategies has also been widely explored to improve the mechanical properties of the 3D-printed constructs. However, this chemical modification is normally recognized as a non-environmentally friendly approach since it involves the use of harmful solvents and reagents as well as laborious procedures, and the use of UV light to promote the crosslinking can affect the viability of laden cells, even though high cell viabilities have been reported in some cases. In fact, several commercially available bioinks are based on methacrylated derivatives of polysaccharides. However, in the future, these toxic reagents or solvents should be avoided, given their recognized danger for living cells, the operator, and the environment. The use of alternative solvent systems, such as ionic liquids (ILs) or deep eutectic solvents (DES), could be considered in the future as a new and safer option for some of these chemical modification approaches.

A different matter of concern when using polysaccharides is their reproducibility and large-scale production. Even though some of them are naturally derived from abundant renewable sources, such as cellulose and chitin, the properties of the polysaccharides are strongly dependent on their sources, and the extraction processes may lead to batch-to-batch variations. This is an issue that should be carefully addressed in the up-scaling studies of promising bioinks. Moreover, regarding the evaluation of the biological properties of the 3D-printed constructs, particularly the post-printing cell viability, diverse time periods (1 day to several days) are considered, which makes it difficult to compare the performance of the bioinks. Some uniformization needs to be considered in future studies.

Nonetheless, the potential of polysaccharides for the development of hydrogel bioinks for 3D bioprinting is undeniable, and the research on this topic is increasing very fast, with new strategies and biomaterials described regularly. In fact, several commercial polysaccharide-based bioinks are already available [208], as highlighted throughout this review, and we foresee that the research and development of new bioinks and strategies will accelerate in the next few years, with new solutions for the market.

## Figures and Tables

**Table 1:** Summary of the recent studies on seaweed derived polysaccharide-based bioinks for 3D bioprinting applications.

Polysaccharide	Other Compounds	Cell Type	Bioink Formulation	Bioprinting Method	Conditions	Construct Properties	Application	Ref.
Alginate	Gelatin Nano-apatite	rBMSC (1 × 10^5^ cells/mL)	Alginate: 6 wt.% Gelatin: 10 wt.% Nano-apatite: 0.1 and 0.5 M Crosslinker: CaCl_2_ 1 M	EB	Nozzle: 0.610 mm Printing speed: 5 mm/s Pressure: 0.5 MPa Temperature: 55 °C	Grid-like scaffolds with 10 × 10 × 5 mm^3^ Compressive strength: 20.7 ± 4.7 to 23.9 ± 1.5 MPa Young’s modulus: 119 ± 26 to 135 ± 36 MPa Cell viability: higher in nano-apatite coated scaffolds with osteogenic differentiation	Bone tissue engineering	[55]
Alginate	Agarose Collagen (Type I)	Primary chondrocytes (1 × 10^7^ cells/mL)	Alginate: 0.1 g/mL Agarose: 15 mg/mL (Alg/Col blend in a ratio of 4:1 and Alg/Agr blend in a ratio of 3:1) Crosslinker: CaCl_2_ 10% (*w*/*v*)	EB	Nozzle: 0.260 mm	Grid-like structures 2 × 2 cm^2^ with 6 layers Compressive modulus: ~50–65 kPa Tensile strength: ~40–45 kPa Cell viability: 95% at day 14 in the Alg/Col blend	Cartilage tissue engineering	[56]
Alginate	--	SK-N-BE cells (1 × 10^7^ cells/mL)	Alginate: 2% (*w*/*v*) Crosslinker: CaCl_2_ in a gelatin support medium	EB	Nozzle: 0.255 mm Printing speed: 8 mm/s Pressure: 12.5 psi	Grid-like geometries Cell viability: 83% at day 7	–	[59]
Alginate	--	RPSCs (2 × 10^5^ cells/mL)	Alginate: 2% (*w*/*v*) engrafted with RGD and YIGSR peptides Crosslinker: CaCl_2_ 50 mM	EB	Nozzle: 0.200 mm Printing speed: 18 mm/s Pressure: 0.3 Bar	Cubic shape scaffold (10 × 10 × 5 mm^3^) with 1 mm distance between strands Young’s modulus: 40.3 ± 2.2, 23.7 ± 3.5, 14.7 ± 3.6, and 14.5 ± 2.7 kPa at day 0, 7, 14, and 21, respectively Cell viability: ~100% at day 7	Nerve tissue engineering	[60]
Alginate	Silk fibroin Pluronic F127	C3A (1 × 10^6^ cells/mL)	Alginate: 5% (*w*/*v*) SF: 5% (*w*/*v*) Pluronic F127: 13% (*w*/*v*) Crosslinker: CaCl_2_ 5% (*w*/*v*)	Co-axial EB	Shell Nozzle: 1.070 mm Core Nozzle: 0.340 mm Printing speed: 15 mm/s Bioink extrusion rate: 7 mL/s Cross-linker solution extrusion rate: 5 mL/s	Grid-like scaffolds with the size of 20 × 20 × 3 mm^3^ Compressive modulus: 16 ± 2.5 kPa Cell viability: ~100% at day 14	–	[61]
Alginate	Gelatin Carbon nanotubes	Fibroblasts (4 × 10^5^ cells/mL)	CNTs: 0.5 and 1%(*w*/*v*) Crosslinker: CaCl_2_	EB	Modified printer for bioprinting of hollow tubular scaffolds Non specified conditions	Circular tubes printed with a 3 mm diameter, an average wall thickness of 0.5 mm and a length of 7–10 cm Young’s modulus: ~1.4 and 0.7 MPa Tensile strength: ~0.9 and 0.5 MPa Cell viability: 85% survival rate until 5 days, with mild toxicity induced by CNTs	Vessels tissue engineering	[62]
Alginate	Albumen (Egg white)	HUVECs (6 × 10^6^ cells/mL)	Alginate: 5% (*w*/*v*) Albumen (egg white) added in volume ratios of 1:1, 2:1, 3:1, 4:1, 5:1 and 6:1. Crosslinker: CaCl_2_	EB	Nozzle: 0.160 mm Pressure: 2.5 Psi CaCl_2_ perfusion bath	Grid structures with 30 × 30 × 1 mm^3^ and 25 × 25 × 1 mm^3^ Cell viability: high up to 5 days with the formation of vascularized channels	–	[63]
Alginate	Albumen (Egg white)	HUVECs (1.25 × 10^6^ cells/mL)	Alginate: 2–3% (*w*/*v*) Dissolved in Albumen (egg white) Crosslinker: CaCl_2_ 500 mM	EB	Nozzle: 0.500 mm Printing speed: 9,10 and 11 mm/s Pressure: 0.3, 0.5 and 0.7 bar	Patches with 12 × 12 mm^2^ and 8 mm height Elastic modulus: 20–27 kPa Cell viability: 94% at day 7	Endothelialized tissue engineering	[64]
Alginate	Collagen type I	hiMPCs (2.5 × 10^6^ to 1 × 10^7^ cells/mL)	Alginate: 2% (*w*/*v*) Collagen: 0.015% (*w*/*v*) With the addition of VEGF growth factor. Crosslinker: CaCl_2_ 20 mM	EB	Nozzle: 0.455 mm Pressure: 100 kPa	Printed spherical discs Cell viability: after 21 days, it was observed the formation of small and large vessels that were transplanted into the chicken embryo chorioallantoic membrane (CAM) model and showed proper blood perfusion	Blood vessels tissue engineering	[65]
Alginate	SFMA	NIH-3T3 (5 × 10^6^ cells/mL)	Alginate: 3 wt.% SFMA: 1,3 and 5 wt.% Crosslinker: CaCO_3_ and UV	EB	Pressure: 10 to 100 kPa Printing speed: 300 to 900 mm/s	Grid like scaffolds with the size of 20 × 20 × 3.5 mm^3^ Young’s modulus decreases with the increments of SFMA concentration. Cell viability: 95% at day 7	–	[66]
Alginate	Hyaluronic acid Gelatin	Mel Im (1 × 10^6^ cells/mL) ADSCs (1 × 10^7^ cells/mL)	Alginate: 0.5% (*w*/*v*) HA: 0.1% (*w*/*v*) Gelatin: 3% (*w*/*v*) Crosslinker: CaCl_2_ 100 mM	EB	Nozzle: 0.580 mm Printing speed: 400 mm/min Pressure: 10–15 kPa	Grids with 1 cm^2^ with 3 layers and 6 strands each Cell viability: ~100% at day 14	In vitro and in vivo metastatic melanoma models	[58]
Alginate	Gelatin DCEL	Primary adult dermal fibroblasts (5 × 10^6^ cells/mL) Primary epidermal keratinocytes (7 × 10^6^ cells/mL)	Alginate: 2% (*w*/*v*) Gelatin: 3.3% (*w*/*v*) DCEL: 0.93% (*w*/*v*) Crosslinker: CaCl_2_ 100 mM	EB	Nozzle: 0.410 mm Pressure: 120 kPa	Three-layered, disc-shaped constructs of 15 mm diameter, about 3 mm height for characterization Rectangular-shaped, single-layered construct (15 mm width, 15 mm length and 1 mm height) for cell culture Young’s modulus: 125 ± 22 kPa Elongation of break: 91.70 ± 9.36% Cell viability: at 21 days of culture, histological analysis showed the formation of both dermal and epidermal equivalent structures	Skin tissue engineering	[57]
Carrageenan	nSi	MC3T3-E1 (N/A)	Carrageenan: 2.5 wt.% nSi: 6 wt.% Crosslinker: CaSO_4_ 1% (*w*/*v*) Gelling temperature: 35 °C	EB	Nozzle: 0.340 mm Printing speed: 4 mm/s Extrusion flow rate: 0.3 mL/h	(i) Single fiber in a lattice network and a layered lattice network; (ii) 30-layer cylinder; (iii) nose and ear models. Compressive modulus: 208 ± 6.5 kPa G’ recovery: 95% Cell viability: 99% at day 7	–	[68]
Carrageenan	nSi GelMA	MC3T3-E1 (1 × 10^6^ cells/mL)	Carrageenan: 1% (*w*/*v*) nSi: 2% (*w*/*v*) GelMA: 10% (*w*/*v*) Crosslinker: KCl and UV	EB	Nozzle: 0.400 mm Printing speed: 20 mm/s Extrusion flow rate: 0.15 mL/h	(i) Single fiber in a lattice network and a layered lattice network; (ii) 30-layer cylinder; (iii) nose and ear models. Compressive modulus: 71.1 ± 4.9 kPa G’ recovery: 75% Cell viability: >90% at day 120	–	[69]
Carrageenan	Gelatin	C2C12 (2.8 × 10^5^ cells/mL in Gel)	Carrageenan: 2% (*w*/*v*) Gel: 8% (*w*/*v*) Gelling temperature: 25 °C	EB	Nozzle: 0.250 mm Temperature: 25 °C	Grid-like scaffolds with 25 × 25 mm^2^ Cell viability: 90% at day 1	–	[71]
Carrageenan	GelMA	C2C12 (3 × 10^5^ cells/mL in Gel MA)	Carrageenan: 2% (*w*/*v*) GelMA: 10% (*w*/*v*) Crosslinker: KCl and UV	EB	Nozzle: 0.250 mm Temperature: 25 °C	Grid-like scaffolds with 25 × 25 mm^2^, line space: 1.3 mm, and 4 layers Cell viability: 96% at day 7	–	[70]
Carrageenan	Alginate	MSCs (5 × 10^5^ cells/mL)	Carrageenan: 1.5% (*w*/*v*) Alginate: 2% (*w*/*v*) Crosslinker: CaSO_4_ 1% (*w*/*v*)	EB	Nozzle: 0.510 mm Printing speed: 2 mm/s Pressure: 50 kPa	Grid-like scaffolds with 25 × 25 mm^2^, line space: 1.3 mm, and 4 layers Storage modulus: 900 Pa Cell viability: higher in Alg-Crg bioinks at day 3	–	[73]
Carrageenan-MA	GelMA	ADSCs (1 × 10^5^ cells/scaffold)	Crg-MA: 1% (*w*/*v*) GelMA: 10% (*w*/*v*) Crosslinker: UV	EB	Nozzle: 0.210 mm Printing speed: 650 mm/min	Grid-like structure with 10 × 10 mm^2^ and 10 layers in height Young’s modulus: 2.2 to 2.5 kPa Cell viability: >80% at day 14	Adipose tissue regeneration	[72]
Carrageenan	nSi GelMA GAG’s proteoglycans	hMSCs	Carrageenan: 1% (*w*/*v*) nSi: 2% (*w*/*v*) GelMA: 7.5% (*w*/*v*) Crosslinker: KCl and UV	EB	Nozzle: 0.400 mm Printing speed: 20 mm/s Extrusion flow rate: 0.15 mL/h	Mandibular models Compressive modulus: 141 ± 8 kPa Cell viability: high with differentiation until day 90 (histological analysis)	Bone tissue engineering	[74]
Agarose	Alginate	Auricular cartilage digested with Collagenase Type 4 cell suspension	Agarose: 2, 3 and 4% (*w*/*v*), combined with alginate in a ratio of 3:2 Gelling temperature: 25 °C	EB	Nozzle: 0.160 mm Pressure: 65–75 Psi	Constructs printed as single lines (print width = 0.5 mm, length = 30 mm) Compressive yield: ~15–20 kPa Cell viability: >~70% cell survival at day 28	Tissue engineering	[75]
Agarose	NOCC	neuro2A (1 × 10^5^ cells/mL)	Agr stock solution: 1% (*w*/*v*) NOOC stock solution: 10% (*w*/*v*) Agr-NOCC 80:20, 60:40, 40:60 and 20:80	EB	Nozzle: 0.410 mm Printing speed: 3 mm/s	Grid-like scaffolds with 20 × 20 × 0.5 mm^3^ Storage modulus: 20 Pa (Agr-NOCC 40:60); 25 Pa (Agr-NOCC 40:60) Printability numbers: 0.95 (Agr-NOCC 40:60); 0.99 (Agr-NOCC 40:60) Cell viability: 100% at day 14 (Agr-NOCC 40:60)	–	[79]

**Abbreviations:** ADSCs–Adipose-derived mesenchymal stem cells; Agr–Agarose; Alg–Alginate; C2C12–Mouse myoblasts cells; C3A–Liver cancer cell line; CNTs–Carbon nanotubes; Col–Collagen; Crg–Carrageenan; Crg-MA–Carrageenan methacrylate; DCEL–Diethylaminoelthyl cellulose; EB–Extrusion bioprinting; G’–storage modulus; Gel–Gelatin; GelMA–Gelatin methacrylate; HA–Hyaluronic acid; hiMPCs–Human induced pluripotent stem cell-derived mesodermal progenitor cells; HUVECs–Human umbilical vein endothelial cells; iPSCs–Induced pluripotent stem cells; MC3T3-E1–Mouse preosteoblasts cell line; Mel Im–Malignant melanoma cell line; MSCs–Mesenchymal stem cells; NOOC–*N*,*O*-Carboxymethyl chitosan; nSi–Nanosilicates; rBMSC–Bone marrow stem cells; RPSCs–Schwann cells; SF–Silk fibroin; SFMA–Silk fibroin methacrylate; UV–Ultraviolet light.

**Table 2:** Summary of the recent studies on higher plants derived polysaccharide-based bioinks for 3D bioprinting applications.

Polysaccharide	Other Compounds	Cell Type	Bioink Formulation	Bioprinting Method	Conditions	Construct Properties	Application	Ref.
CMC	Sodium alginate	Human pancreatic cancer cells (2 × 10^6^ cells/mL)	Alginate: 4% (*w*/*v*) Alg:CMC: 4:1, 2:1, 4:3 and 1:1 (dry mass) Crosslinker: CaCl_2_ 4% (*w*/*v*)	EB	Nozzle: 0.410 mm Printing speed: 5 mm/s Pressure: 8 psi	Cubic model (10 × 10 × 2 mm^3^ with 1 mm of filament distance) was printed Young’s modulus: >75 kPa for >4% CMC Cell viability: >70% for alginate/CMC, for 15 and 23 days after bioprinting	–	[100]
Sodium carboxymethyl cellulose methacrylate	GelMA, AlgMA PEGDA	C2C12 (1 × 10^7^ cells/mL)	GelMA: 1 or 5% (*w*/*v*) CMCMA, AlgMA, or PEGDA: 1% (*w*/*v*) Crosslinker: UV	EB	Nozzle: 0.200 mm Printing speed: 7 mm/s Pressure: 2.5 bar Temperature: 10 °C	Cylindrical model (10 mm in diameter) Compressive modulus: 1.96 ± 0.16 kPa (GelMA-CMCMA) Cell viability: 60%	Muscle tissue engineering	[109]
Methyl cellulose	Alginate	Bovine primary chondrocytes (5 × 10^6^ cells/g)	Alginate: 3 wt.% MC: 9 wt.% Crosslinker: CaCl_2_ 100 mM	EB	Nozzle: 0.610 mm Printing speed: 10 mm/s Pressure: 70–80 kPa	Cubic model (9.5 × 9.5 × 1.4 mm^3^) Compressive strength: 45.2 ± 8.0 MPa for UV-treated, 32.1 ± 6.8 MPa for the autoclaved, and 27.7 ± 4.6 MPa for scCO_2_Cell viability: >50% for all samples, except for scCO_2_-treated	–	[101]
NorCMC and cCMC	N/A	hMSCs, NIH 3T3 and HUVECs (1 × 10^7^ cells/mL)	cCMC: 15% (*w*/*v*) NorCMC: 10% (*w*/*v*) Thiol: norbornene: to 1:4, 1:2 and 1:1. Crosslinker: UV	EB	Printing speed: 5–10 mm/s Increased pressure from 1368 kPa (30 min) to 276 kPa (60 min) and 345 kPa (90 min)	Grid-like construct (15 × 15 mm^2^) Compression modulus: 46 to 316 kPa when increasing from 1:4 to 1:2, for cCMC, and from 40 to 133 kPa for NorCMC Cell viability: >80% for all cell lines	–	[111]
Hydroxyethyl cellulose	Sodium alginate, Gelatin	MCF-7 (10^7^ cells/mL)	Sodium alginate: 1% (*w*/*v*) Gelatin: 5% (*w*/*v*) Hydoxyethyl cellulose: 1% (*w*/*v*) Crosslinker: CaCl_2_ 1.5% (*w*/*v*)	EB	Printing speed: 5 mm/s Temperature: 25ºC	Cylindrical model (9 × 8 mm^2^); spheroid model and human ear structure Compressive modulus: 13 kPa for hydroxyethyl cellulose-reinforced constructs Cell viability: 98%	Breast tumor model	[112]
Hydroxypropyl methyl cellulose-Si	NaF and glycine	hMSCs (1.106 cells/mL)	NaF and/or glycine was added to obtain a final HPMC-Si concentration of 135 g/L Gelation temperature: room temperature	EB	Nozzle: 0.210 mm Printing speed: 10 mm/s Pressure: 3 bar Temperature: 37 °C	Grid-like structures Young’s modulus: 99 ± 15 kPa Cell viability: LIVE/DEAD assay indicated that 3D bioprinting did not affect cells since most green living cells were observed at day 1 and till day 7	–	[113]
Hydroxypropyl methyl cellulose methacrylate	Silk fibroin	BMSCs (1 × 10^6^ cells/mL)	Hydroxypropyl methyl cellulose methacrylate: 5 wt.% Silk fibroin: Hydroxypropyl methyl cellulose methacrylate: 4:0, 3:1, 2:2, 1:4 and 0:4 Crosslinker: UV	EB	Nozzle: 0.160 mm Printing speed: 20 mm/s Pressure: 30–80 kPa	Ring-like structure (8 mm diameter); cylindrical (8 × 4 mm^2^) open structure and human ear structure Compressive stress: above 100 kPa for proportion 3:1 Cell viability: nonnegligible cell dead 46% at day 1 and decreased to 3% at day 10	Cartilage tissue repair	[114]
NFC	Poly(2-ethyl-2-oxazoline), Sortase A and alginate	hACs (10^7^ cells/mL)	Poly(2-ethyl-2-oxazoline): 5% (*w*/*v*) Alginate: 5% (*w*/*v*) NFC: 0.5, 1.0, 1.5 and 2.0% (*w*/*v*) Crosslinker: Sortase A 100 µM and CaCl_2_ 10 mM	EB	Nozzle: 0.410 mm Pressure: 18–21 kPa	Grid-like structures Compressive modulus: ~30 kPa Cell viability: 90 ± 2%	Cartilage tissue engineering	[115]
NFC	Horseradish peroxidase, glucose, and alginate	10T1/2 (5 × 10^5^ cells/mL)	NFC: 0.5–1.5% (*w*/*v*) Alginate: 0.5% (*w*/*v*) Crosslinker: horseradish peroxidase 100 (units/mL)	EB	Nozzle: 0.210 mm Printing speed: 22 mm/s	Lattice structure (20 × 21 mm^2^) and human nose (12 × 15 mm^2^) Cell viability: 54.1 ± 0.6% at day 1 and 56.0 ± 2.4% at day 7	–	[116]
NFC	Alginate, CMC	hSF (10^6^ cells/mL)	Alginate: 3 wt.% CMC: wt. 3% NFC: 1.5 wt.% Crosslinker: CaCl_2_ 2 wt.%	EB	Nozzle: 0.250 mm	Cylinder-shaped structure (10 × 0.8 mm^2^) Cell viability: LIVE/DEAD assay indicated a homogeneous cell distribution	In vitro model of the human dermis	[117]
NFC	Alginate	hMFC (10^7^ cells/mL)	NFC:Alg: 01:00, 20:80, 50:50, 60:40, 70:30, 80:20 and 90:10, with a solid content of 3.5% (*w*/*v*) Crosslinker: CaCl_2_ 100 mM	EB	Nozzle: 0.413 mm Printing speed: 10 mm/s Pressure: 55–200 kPa	Block (20 × 20 × 3 mm^3^) Peak modulus <10 kPa for the 10–40% cumulative strain Cell viability: >60%	Human meniscus tissue engineering	[118]
NFC	Alginate and polydopamine nanoparticles	MC3T3-E1 (6 × 10^3^ cells/cm^2^)	Alginate: 2.1, 1.5 and 0.9% (*w*/*v*) NFC: 2.1, 1.5 and 0.9% (*w*/*v*) Polydopamine nanoparticles: 0.5% (*w*/*v*) Crosslinker: CaCl_2_ 5% (*w*/*v*)	EB	Nozzle: 0.500 mm Printing speed: 5 mm/s	Grid structure (20 × 20 mm^2^) Compressive modulus: 2.03 ± 0.31 kPa (higher for 1.5% (*w*/*v*) of alginate and NFC with 0.5% (*w*/*v*) polydopamine nanoparticles) Cell viability: >75%	Bone tissue engineering	[102]
NFC	Alginate and fibrinogen	C2C12 (25 × 10^6^ cells/mL)	Commercial inks: gelatin methacrylate and alginate crosslinked by UV light (CELLINK^®^ GelMA A); (2) gelatin methacrylate, xanthan gum, and alginate- fibrinogen (CELLINK^®^ GelXA FIBRIN); (3) nanofibrillated cellulose (NFC)/alginate-fibrinogen crosslinked with CaCl_2_ and thrombin (CELLINK ^®^ FIBRIN)	EB	Nozzle: 0.250 mm Printing speed: 16 mm/s Pressure: 10–15 kPa	Lines (length: 20 mm and thickness: 0.35 mm) Cell viability: >90%	Skeletal muscle regeneration	[103]
CNC	Platelet lysate	hASCs (1 × 10^6^ cells/mL)	Aldehyde CNC: 18 wt.% Platelet lysate: 2.88 wt.% Crosslinker: CaCl_2_ 10 mM	EB	Nozzle: 0.210 mm Printing speed: 5 mm/s Temperature: 20 °C	Square lattice (1 × 1 × 0.25 cm^2^) Cell viability: >90%	–	[104]
CNC	Gelatin methacryloyl and hyaluronic acid methacrylate	ATDC5 (1 × 10^6^ cells/mL)	CNC: 1, 5, 10 and 15% (*w*/*v*) GelMA: 10% (*w*/*v*) HAMA: 2% (*w*/*v*) Crosslinker: UV	EB	Nozzle: 0.200 mm Printing speed: 8–12 mm/s Pressure: 2–4 bar	Cuboid structures (10 × 10 × 1.5 mm^3^) Compressive modulus: 22.7 ± 2.8 kPa to 55.8 ± 2.1 kPa with increasing CNC loading to 10% (*w*/*v*) Cell viability: >90% until 7 days after bioprinting	–	[110]
CNC	Chitosan, hydroxyethyl cellulose	MC3T3-E1 (5 × 10^6^ cells/mL)	Chitosan: 3% (*w*/*v*) Hydroxyethyl cellulose (0–0.5 mg/mL) CNC: 0–2% (*w*/*v*) Crosslinker: β-glycerophosphate 100 mM and	EB	Nozzle: 0.900 mm Printing speed: 2 mm/s Pressure: 20 kPa	Cylindrical scaffolds (7.5 × 4 mm^2^) Young’s modulus: 85.12 ± 4.31 Pa for chitosan, to 122.12 ± 13.84 Pa for 0.5% CNC and to 132.40 ± 2.55 Pa for 1.5% CNC Cell viability: qualitative analysis through LIVE/DEAD indicated that bioprinting cell-laden bioinks did not comprise cell viability	–	[105]
CNC	k-carrageenan and methylcellulose	L929 (3 × 10^5^ cells/mL)	k-carrageenan: 0.3 wt.% Methylcellulose: 7 wt.% CNC: 2 or 4 wt.%	EB	Nozzle: 0.200 mm Printing speed: 1 mm/s Pressure: 110 kPa Temperature: 25 °C	Grid-like constructs (10 × 10 cm^2^) Compressive stress: 20.03 ± 0.02 and 23.28 ± 0.01 kPa when increasing CNC content from 2 to 4 wt.% Cell viability: >90%	–	[106]
BC	Alginate and GelMA	RSC96 (15 × 10^6^ cells/mL)	Alginate: 5% (*w*/*v*) GelMA: 5% (*w*/*v*) BC: 0.3% (*w*/*v*) Crosslinker: CaCl_2_ 50 mM and blue light	EB	Nozzle: 0.160 mm Printing speed: 30 mm/s Temperature: 20–25 °C	Cuboid structure (8 × 8 × 2 mm^3^), cylinder (5 × 4 mm^2^) Compressive modulus: 2.25 kPa to 10.92 kPa with the incorporation of BC Cell viability: the addition of BC did not affect cell proliferation as cell proliferation absorbance increase to > 3 at day 7	–	[107]
TEMPO oxidized bacterial NFC	N/A	R1/E (3 × 10^7^ cells/mL)	TEMPO oxidized bacterial NFC: 1% (*w*/*v*) Crosslinker: N/A	EB	Nozzle: 0.900 mm Room temperature	Grid-like constructs Cell viability: LIVE/DEAD assay showed that only at day 7 cells started to stretch and elongate	–	[108]
Pectin methacrylate	N/A	Human neonatal dermal fibroblasts (1.5 or 2.5 wt.%)	Pectin methacrylate: 1.5–2.5 wt.% Crosslinker: UV	EB	Nozzle: 0.642 mm	Cuboid structures (8 × 8 × 4.5 mm^3^ and 17 × 17 × 2.4 mm^3^) Cell viability: LIVE/DEAD showed that after 24 h post-printing the printed constructs displayed viable cells	Dermal tissue engineering	[119]
Pectin	Pluronic F127 and alginate	MIN6 (1 × 10^7^ cells/mL)	Pectin: 2 wt.% Alginate: 6 wt.% Pluronic F127: 8 wt.% Crosslinker: CaCl_2_ 5 mM	EB	Nozzle: 0.455 mm Printing speed: 4 mm/s Pressure: 50 psi Temperature: 30 ± 3 °C	Grid-like structures (8 × 2 mm^2^) Cell viability: ≥80 ± 3.7% during the 7 days of culture	–	[120]

**Abbreviations:** 10T1/2–Mouse fibroblasts; Alg–Alginate; AlgMA–Alginate methacrylate; ATDC5-Mouse teratocarcinoma cells; BC–Bacterial cellulose; BMSCs–Bone marrow mesenchymal stem cells; C2C12-Mouse myoblasts cells; cCMC-Carbic (norbornene) functionalized CMC; CMC–Carboxymethyl cellulose, CMCMA–Carboxymethyl cellulose methacrylate; CNC–Cellulose nanocrystals; GelMA–Gelatin methacrylate; hACs–Human auricular chondrocytes; HAMA–Hyaluronic acid methacrylate; hASCs–Human adipose-derived stem cells; hMFC–Human meniscus fibrochondrocytes; hMSCs–Human mesenchymal stromal cells; hSF–Human-derived skin fibroblasts; HPMC–Hydroxypropyl methyl cellulose; HUVECs-Human umbilical vein endothelial cells; L929–Mouse fibroblasts; MC–Methyl cellulose; MC3T3-E1–Pre-osteoblasts; MCF-7–Human breast cancer cell line; MIN6–Mouse insulinoma cells; NIH3T3–Fibroblasts cell line; NFC– Nanofibrillated cellulose; NorCMC–Norbornene CMC; R1/E–Pluripotent mouse embryonic stem cells; PEGDA-Poly(ethylene glycol) diacrylate RSC96–Schwann cells; TEMPO-(2,2,6,6-Tetramethylpiperidin-1-yl)oxyl.

**Table 3:** Summary of the recent studies on microbial derived polysaccharide-based bioinks for 3D bioprinting applications.

Polysaccharide	Other Compounds	Cell Type	Bioink Formulation	Bioprinting Method	Conditions	Construct Properties	Application	Ref.
Dextran	GelMA, succinylated chitosan and dextran aldehyde	hBMSC (1.0 × 10^6^ cells/mL) and HUVEC (1.0 × 10^6^ cells/mL)	Succinylated chitosan: 8% (*w*/*v*) Dextran aldehyde: 0.6% (*w*/*v*) GelMA: 13% (*w*/*v*) Crosslinker: UV	EB	Nozzle: 0.400 mm Printing speed: 5 mm/s Pressure: 150–225 kPa Temperature: 25 °C	Core/shell structure (12 × 12 × 4 mm^3^) Young’s modulus: 100 kPa for GelMA and 50 kPa for chitosan-dextran hydrogel Cell viability: cell growth increased until day 21	Wound healing	[151]
Xanthan gum	GelMA, alginate and CMC	hMSCS (2.5 × 10^6^ cells/mL)	GelMA: 10% (*w*/*v*) Alginate: 2% (*w*/*v*) CMC: 1 or 2% (*w*/*v*) Xanthan gum: 1 or 2% (*w*/*v*) Crosslinker: UV and CaCl_2_	EB	Nozzle: 0.515 mm	Grid-like structures Young’s modulus: >40 kPa for UV + ionic (with Ca^2+^)-crosslinked hydrogels, >20 kPa for UV-crosslinked and <20 kPa ion-crosslinked Cell viability: >80%	–	[152]
Xanthan gum	Collagen type 1	ECs/hESC-ECs (10 × 10^6^ cells/mL) and hESC-FBs (2 × 10^6^ cells/mL)	Xanthan gum: 10% (*w*/*v*) Collagen: 4.73 mg/mL	EB	Nozzle: 0.410 mm Printing speed: 15 mm/s Pressure: 29–35 kPa	Grid-like constructures (10 ×10 × 3 mm^3^) Cell viability: 92.39 ± 2.02% at 24 h post printing and 89.40 ± 2.58% at 48 h post printing	–	[155]
Xanthan gum	Gelatin	Primary human-derived-skin fibroblasts (0.5 × 10^6^ cells/mL) and HaCaTs (5 × 10^6^ cells/mL)	Xanthan gum: 0.3, 0.7 1 and 1.2% (*w*/*v*) Gelatin: 2.5 and 3% (*w*/*v*) Crosslinker: glutaraldehyde 0.3, 0.5 and 1% (v/v)	EB	Nozzle: 0.250 mm Pressure: 10–20 kPa	Grid-like constructs (1 cm^2^) Cell viability: bioprinting process did not affect the cell viability, as no sign of cell death was visible on day 1 after bioprinting	–	[154]
Gellan gum	Poly(lactic acid), GelMA	Mesenchymal stromal cells (10 × 10^6^ cells/mL)	Gellan gum: 1% (*w*/*v*) GelMA: 10% (*w*/*v*) Crosslinker: UV	EB	Nozzle: 0.908 mm Printing speed: 7.9 mm/s Temperature: room temperature	Grid-like structures (2.25 mm line spacing) Cell viability: >80% after 3 days	–	[156]
Gellan gum modified with RGD	N/A	Primary cortical neurons (1 × 10^6^ cells/mL)	RGD-gellan gum: 1% (*w*/*v*) Crosslinker: CaCl_2_ 1 M	EB	Nozzle: 0.200 mm	Cylindrical structure Cell viability: >70% until day 7 after bioprinting.	–	[157]
Gellan gum	PEGDA	BMSCs (2 × 10^6^ cells/mL)	Gellan gum: 0.75 wt.% PEGDA: 15 wt.% Crosslinker: UV	EB	Nozzle: 0.515 mm Temperature: 37 °C	Rectilinear and honeycomb structures Young’s modulus: higher values for honeycomb structures than for rectilinear Cell viability: >90%	Intervertebral disc regeneration	[158]
Gellan gum	PEGDA	BMSCs and MC3T3-E1 (2 × 10^6^ cells/mL)	Gellan gum: 1.0, 1.5, 2.0 wt.% PEGDA: 0, 5.0, 10.0 and 15.0 wt.% Crosslinker: UV	EB	Nozzle: 0.515 mm Printing speed: 10 mm/s Temperature: 37 °C	Sharp cone (10 mm in diameter and height), square prism (bottom diameter 10 mm, top diameter 10 mm, height 10 mm) and human scale ear and nose Young’s modulus: UV crosslinking of G1.5P10 (chosen formulation) caused an improvement in the Young’s modulus from 40 kPa to 60 kPa Cell viability: >87%	–	[159]
Gellan gum	GelMA	C2C12 (4 × 10^6^ cells/mL)	GelMA: 2, 4, 10, 15, 20, 30% (*w*/*v*) Gellan gum: 0, 0.2, 0.4, 1, 1.5% (*w*/*v*) Crosslinker: UV	EB	Nozzle: 0.410 mm Printing speed: 1.7 mm/s Pressure: 1.2 bar Temperature: 25 °C	Grid pattern (9 × 9 × 10 mm^3^) and tubular structure Compressive modulus: 9–16 kPa Cell viability: maintained > 95% at all time points (0, 7 and 14 days).	Soft tissue engineering	[160]
Gellan gum	Sodium alginate and thixotropic magnesium phosphate-based gel	MG-63 (1 × 10^6^ cells/mL)	Sodium alginate: 2.5 or 4.0% (*w*/*v*) Gellan gum: 3.0 or 2.0% (*w*/*v*) Sodium alginate-gellan gum to thixotropic magnesium phosphate-based gel (1.5:1) Crosslinker: UV	EB	Nozzle: 0.410 mm Printing speed: 0.005 mL/s	Grid-like constructs (20 × 20 mm^3^), human mandible, university symbol abbreviation and human nose Compressive stiffness: 299 ± 71 kPa for 2.0% gellan gum and 4.0% (*w*/*v*) sodium alginate Cell viability: relative proliferation rate >100% 5 and 7 days after bioprinting	Osteochondral repair	[161]
Gellan gum	Fibrinogen	pMCs (1.5 × 10^7^ cells/mL)	Gellan gum: 12 mg/mL Fibrinogen: 25, 50, 75, 100, 125, and 150 mg/mL Crosslinker: thrombin 20 (U/mL) and UV	EB	Nozzle: 0.240 mm Printing speed: 250 mm/min Pressure: 45–65 kPa	Cuboid structures (10 × 10 × 5 mm^3^) Compressive elastic modulus: ink with 100 mg/mL of fibrinogen had the highest with values increasing from 13.6 ± 1.5 to 23.1 ± 2.7 KPa at 3% strain, 14.8 ± 2.1 to 24.3 ± 1.8 KPa at 6% strain and 17.9 ± 3.2 to 27.5 ± 2.3 KPa at 12% Cell viability: >90% during the culture	Fibrocartilaginous tissue regeneration	[162]
Gellan gum	Alginate and laminin	hiNPCs	Two different alginate-gellan gum blends were prepared: 1.5% (*w*/*v*) alginate, 0.5% (*w*/*v*) gellan gum and 0.01% (*w*/*v*) laminin, and 0.3% (*w*/*v*) alginate, 0.8% (*w*/*v*) and 0.01% (*w*/*v*) laminin gellan gum Crosslinker: CaCl_2_ 0.09 M	EB	Nozzle: 0.200 mm Printing speed: 4.1 mm/s Pressure: 450–550 kPa	Grid-like structures Elastic modulus: 0.3% Alg-0.8% gellan gum-0.01% laminin had the lowest elastic modulus (20 kPa) when compared to 1.5% Alg-0.5% gellan gum-0.01% laminin (35 kPa) Cell viability: 60%	In vitro neural models	[153]

**Abbreviations:** BMSCs–Bone marrow mesenchymal stem cells; C2C12–Mouse myoblasts cells; CMC–Carboxymethyl cellulose; ECs/hESC-ECs–Human embryonic stem cells and endothelial cells; GelMA–Gelatin methacrylate; HaCaTs–Human epidermal keratinocyte; hBMSCs–Human bone marrow derived mesenchymal stem cells; hINPC-hMSCs–Human mesenchymal stromal cells; HUVECS–Human umbilical vein endothelial cells; hINPC–Human induced pluripotent stem cells-derived neural progenitor cells; MC3T2-E1–Pre-osteoblasts; MG-63–Osteosarcoma cells; pMCs–Porcine primary meniscus cells.

**Table 4:** Summary of recent works on chitosan-based bioinks for 3D bioprinting applications.

Polysaccharide	Other Compounds	Cell Type	Bioink Formulation	Bioprinting Method	Conditions	Construct Properties	Application	Ref.
Chitosan	D-(+)-raffinose pentahydrate	Primary human skin fibroblasts	Chitosan: 6% (*w*/*v*) D-(+)-raffinose pentahydrate: 290 mM Crosslinkers: KOH 1.5 M Na_2_CO_3_ 1.5 M, ammonia vapours	EB	Nozzle: 0.260 mm Printing speed: 3 mm/s	Grid structure with 1.6 × 1.6 cm^2^ Young’s modulus: KOH: 105 kPa ± 18 kPa; Na_2_CO_3_: 94 kPa ± 19 kPa; ammonia vapours 128 kPa ± 21 kPa Cell viability: enhanced cell growth up to 21 days	–	[190]
Chitosan	-	PDLSCs (5 × 10^5^ cells/mL)	Chitosan: 1.67% (*w*/*v*) Crosslinker: K_2_HPO_4_ and NaHCO_3_	EB	N/A	Lattice-type structure (thickness of 2 mm × 8-layer height) Cell viability: high viability for 7 days.	–	[191]
Chitosan methacrylate	LAP	HUVECs (1 × 10^6^ cells/mL)	Chitosan: 1% (*w*/*v*) LAP: 0.2 wt.% Crosslinker: UV	EB/DLP	DLP photocuring conditions: 405 nm, 15 mW/cm^2^, 15 s	Lattice structure with 10 × 10 × 1 mm^3^ Compressive modulus: Increased from 315 kPa to 910 kPa, with CSMA.	Tissue engineering	[192]
Carboxymethyl chitosan	Oxidized and non-oxidized hyaluronic acid	L929 (1 × 10^6^ cells/mL)	Carboxymethyl chitosan: 2 wt.% Hyaluronic acid: 0.4 wt.% Oxidized hyaluronic acid: 4 wt.% Crosslinker: FeCl_3_ 20 mM	EB	Nozzle: 0.200 mm Printing speed: 5–25 mm/s	2- and 4-layered grid square scaffolds (12 × 12 mm^2^ printed area) Cell viability: 96% at day 7 and 95% at day 14	–	[193]
Chitosan	Gelatin PEG-Star-Ma	U87 (7 × 10^5^ cells/mL)	Chitosan/Gelatin/PEG-Star-ma ratio = 1:3:0.05% (*w*/*v*) Gelling temperature: 37 °C	EB	Nozzle: 0.410 mm Pressure: 25–35 kPa Temperature: 37 °C	Grid-like structure Cell viability: maintained for 6 days	In vitro models	[194]
Chitosan	PEG, α-cyclodextrin and gelatin	MSCs (1 × 10^7^ cells/mL)	CS-PEG at 30 mg.mL. Crosslinker: β-glycerophosphate	EB	Nozzle: 0.300–0.400 mm	3D columnar structures (10 mm diameter × 3 mm thickness) Young’s modulus: 4 kPa to 130 kPa MSCs differentiated better towards adipose cells in bioink with a Young’s modulus of 60 kPa, while bioinks with 10–20 kPa favoured the MSCs differentiation towards neuron-like cells.	–	[195]
Chitosan	Gamma-PGA	Human adult fibroblasts (2 × 10^5^ cells/mL)	Chitosan: 4.5% and 6% (*w*/*v*) Gamma-PGA: 2–20% (*w*/*v*) Gelling temperature: 37 °C	EB	Nozzle: 0.700 mm (CS) 0.500 mm (Gamma-PGA) Pressure: 25–40 kPa (CS) 5–10 kPa (Gamma-PGA) Temperature: 37 °C Printing speed: 10 mm/s	Rectangular grid structure with 20 × 10 × 1.2 mm^3^ Cell viability: ~70% after 24 h	–	[196]
Chitosan methacrylate	β-glycerol phosphate salt	NIH 3T3 (1 × 10^6^ cells/mL)	Chitosan: 1.5% (*w*/*v*) Crosslinker: UV	EB	Nozzle: 0.720, 0.510 and 0.410 mm Temperature: 37 °C	Grid-like structure The developed hydrogel was non-cytotoxic After bioprinting, NIH 3T3 cells were well dispersed and proliferation was observed.	–	[197]

**Abbreviations:** CS–Chitosan; CSMA–Chitosan methacrylate; DLP–Digital light processing; EB–Extrusion bioprinting; L929–Mouse fibroblasts cell line; Gamma-PGA–Poly-gamma-glutamic acid: LAP–Lithium phenyl-2,4,6-trimethylbenzoylphosphinate; MSCs–Mesenchymal stem cells; NIH 3T3–Mouse fibroblasts cell line; PDLSCs–Periodontal ligament stem cells; PEG–poly(ethylene glycol); PEG-Star-ma–Poly(ethylene glycol)-Star-maleimide; HUVECs–Human umbilical vein endothelial cells; U87–Malignant glioma cell line; UV–Ultraviolet light.

## References

[1] Hong N., Yang G.-H., Lee J., Kim G. (2018). 3D Bioprinting and Its in Vivo Applications. J. Biomed. Mater. Res. Part B Appl. Biomater..

[2] Burke M., Carter B.M., Perriman A.W. (2017). Bioprinting: Uncovering the Utility Layer-by-Layer. J. 3D Print. Med..

[3] Chen X., Naghieh S. (2019). Extrusion Bioprinting of Scaffolds. Extrusion Bio-Printing of Scaffolds for Tissue Engineering Applications.

[4] Kumar P., Ebbens S., Zhao X. (2021). Inkjet Printing of Mammalian Cells—Theory and Applications. Bioprinting.

[5] Zennifer A., Subramanian A., Sethuraman S. (2022). Design Considerations of Bioinks for Laser Bioprinting Technique towards Tissue Regenerative Applications. Bioprinting.

[6] Zennifer A., Manivannan S., Sethuraman S., Kumbar S.G., Sundaramurthi D. (2022). 3D Bioprinting and Photocrosslinking: Emerging Strategies & Future Perspectives. Biomater. Adv..

[7] Ozbolat I.T., Peng W., Ozbolat V. (2016). Application Areas of 3D Bioprinting. Drug Discov. Today.

[8] Matai I., Kaur G., Seyedsalehi A., McClinton A., Laurencin C.T. (2020). Progress in 3D Bioprinting Technology for Tissue/Organ Regenerative Engineering. Biomaterials.

[9] Koçak E., Yıldız A., Acartürk F. (2021). Three Dimensional Bioprinting Technology: Applications in Pharmaceutical and Biomedical Area. Colloids Surfaces B Biointerfaces.

[10] Tiwari A.P., Thorat N.D., Pricl S., Patil R.M., Rohiwal S., Townley H. (2021). Bioink: A 3D-Bioprinting Tool for Anticancer Drug Discovery and Cancer Management. Drug Discov. Today.

[11] Williams D., Thayer P., Martinez H., Gatenholm E., Khademhosseini A. (2018). A Perspective on the Physical, Mechanical and Biological Specifications of Bioinks and the Development of Functional Tissues in 3D Bioprinting. Bioprinting.

[12] Skardal A., Atala A. (2015). Biomaterials for Integration with 3-D Bioprinting. Ann. Biomed. Eng..

[13] Liu F., Liu C., Chen Q., Ao Q., Tian X., Fan J., Tong H., Wang X. (2018). Progress in Organ 3D Bioprinting. Int. J. Bioprinting.

[14] Vijayavenkataraman S., Yan W.-C., Lu W.F., Wang C.-H., Fuh J.Y.H. (2018). 3D Bioprinting of Tissues and Organs for Regenerative Medicine. Adv. Drug Deliv. Rev..

[15] Hospodiuk M., Dey M., Sosnoski D., Ozbolat I.T. (2017). The Bioink: A Comprehensive Review on Bioprintable Materials. Biotechnol. Adv..

[16] Suntornnond R., An J., Chua C.K. (2017). Roles of Support Materials in 3D Bioprinting. Int. J. Bioprinting.

[17] Norotte C., Marga F.S., Niklason L.E., Forgacs G. (2009). Scaffold-Free Vascular Tissue Engineering Using Bioprinting. Biomaterials.

[18] Unagolla J.M., Jayasuriya A.C. (2020). Hydrogel-Based 3D Bioprinting: A Comprehensive Review on Cell-Laden Hydrogels, Bioink Formulations, and Future Perspectives. Appl. Mater. Today.

[19] Mahinroosta M., Jomeh Farsangi Z., Allahverdi A., Shakoori Z. (2018). Hydrogels as Intelligent Materials: A Brief Review of Synthesis, Properties and Applications. Mater. Today Chem..

[20] Bi X., Liang A., Majee A.L.E.-S.B. (2016). In Situ-Forming Cross-linking Hydrogel Systems: Chemistry and Biomedical Applications. Emerging Concepts in Analysis and Applications of Hydrogels.

[21] Silva N.H.C.S., Vilela C., Marrucho I.M., Freire C.S.R., Pascoal Neto C., Silvestre A.J.D. (2014). Protein-Based Materials: From Sources to Innovative Sustainable Materials for Biomedical Applications. J. Mater. Chem. B.

[22] Reddy N., Reddy R., Jiang Q. (2015). Crosslinking Biopolymers for Biomedical Applications. Trends Biotechnol..

[23] Silva A.C.Q., Silvestre A.J.D., Vilela C., Freire C.S.R. (2021). Natural Polymers-Based Materials: A Contribution to a Greener Future. Molecules.

[24] Stanton M.M., Samitier J., Sánchez S. (2015). Bioprinting of 3D Hydrogels. Lab Chip.

[25] Hassan M., Dave K., Chandrawati R., Dehghani F., Gomes V.G. (2019). 3D Printing of Biopolymer Nanocomposites for Tissue Engineering: Nanomaterials, Processing and Structure-Function Relation. Eur. Polym. J..

[26] Diekjürgen D., Grainger D.W. (2017). Polysaccharide Matrices Used in 3D in Vitro Cell Culture Systems. Biomaterials.

[27] Singh M.R., Patel S., Singh D. (2016). Natural Polymer-Based Hydrogels as Scaffolds for Tissue Engineering. Nanobiomaterials in Soft Tissue Engineering.

[28] Mohan T., Maver T., Štiglic A.D., Stana-Kleinschek K., Kargl R., Thomas S., Balakrishnan P., Sreekala M.S.B.T.-F.B.P. (2018). 3D Bioprinting of Polysaccharides and Their Derivatives: From Characterization to Application. Fundamental Biomaterials: Polymers.

[29] Axpe E., Oyen M. (2016). Applications of Alginate-Based Bioinks in 3D Bioprinting. Int. J. Mol. Sci..

[30] Piras C.C., Smith D.K. (2020). Multicomponent Polysaccharide Alginate-Based Bioinks. J. Mater. Chem. B.

[31] Petta D., D’Amora U., Ambrosio L., Grijpma D.W., Eglin D., D’Este M. (2020). Hyaluronic Acid as a Bioink for Extrusion-Based 3D Printing. Biofabrication.

[32] Wang X., Wang Q., Xu C. (2020). Nanocellulose-Based Inks for 3D Bioprinting: Key Aspects in Research Development and Challenging Perspectives in Applications—A Mini Review. Bioengineering.

[33] Benwood C., Chrenek J., Kirsch R.L., Masri N.Z., Richards H., Teetzen K., Willerth S.M. (2021). Natural Biomaterials and Their Use as Bioinks for Printing Tissues. Bioengineering.

[34] Decante G., Costa J.B., Silva-Correia J., Collins M.N., Reis R.L., Oliveira J.M. (2021). Engineering Bioinks for 3D Bioprinting. Biofabrication.

[35] Ganpisetti R., Lalatsa A. (2021). Cellulose Bio–Ink on 3D Printing Applications. J. Young Pharm..

[36] Khoeini R., Nosrati H., Akbarzadeh A., Eftekhari A., Kavetskyy T., Khalilov R., Ahmadian E., Nasibova A., Datta P., Roshangar L. (2021). Natural and Synthetic Bioinks for 3D Bioprinting. Adv. NanoBiomed Res..

[37] Mahendiran B., Muthusamy S., Sampath S., Jaisankar S.N., Popat K.C., Selvakumar R., Krishnakumar G.S. (2021). Recent Trends in Natural Polysaccharide Based Bioinks for Multiscale 3D Printing in Tissue Regeneration: A Review. Int. J. Biol. Macromol..

[38] Moghaddam A.S., Khonakdar H.A., Arjmand M., Jafari S.H., Bagher Z., Moghaddam Z.S., Chimerad M., Sisakht M.M., Shojaei S. (2021). Review of Bioprinting in Regenerative Medicine: Naturally Derived Bioinks and Stem Cells. ACS Appl. Bio Mater..

[39] Pedroza-González S.C., Rodriguez-Salvador M., Pérez Benítez B.E., Alvarez M.M., Trujillo-de Santiago G. (2021). Bioinks for 3D Bioprinting: A Scientometric Analysis of Two Decades of Progress. Int. J. Bioprinting.

[40] Piras C.C., Fernández-Prieto S., De Borggraeve W.M. (2017). Nanocellulosic Materials as Bioinks for 3D Bioprinting. Biomater. Sci..

[41] Saddique A., Cheong I.W. (2021). Recent Advances in Three-Dimensional Bioprinted Nanocellulose-Based Hydrogel Scaffolds for Biomedical Applications. Korean J. Chem. Eng..

[42] Tarassoli S.P., Jessop Z.M., Jovic T., Hawkins K., Whitaker I.S. (2021). Candidate Bioinks for Extrusion 3D Bioprinting—A Systematic Review of the Literature. Front. Bioeng. Biotechnol..

[43] Parimala Chelvi Ratnamani M., Zhang X., Wang H. (2022). A Comprehensive Assessment on the Pivotal Role of Hydrogels in Scaffold-Based Bioprinting. Gels.

[44] Taghizadeh M., Taghizadeh A., Yazdi M.K., Zarrintaj P., Stadler F.J., Ramsey J.D., Habibzadeh S., Hosseini Rad S., Naderi G., Saeb M.R. (2022). Chitosan-Based Inks for 3D Printing and Bioprinting. Green Chem..

[45] Zhou K., Sun Y., Yang J., Mao H., Gu Z. (2022). Hydrogels for 3D Embedded Bioprinting: A Focused Review on Bioinks and Support Baths. J. Mater. Chem. B.

[46] Sultan S., Siqueira G., Zimmermann T., Mathew A.P. (2017). 3D Printing of Nano-Cellulosic Biomaterials for Medical Applications. Curr. Opin. Biomed. Eng..

[47] Athukoralalage S.S., Balu R., Dutta N.K., Choudhury N.R. (2019). 3D Bioprinted Nanocellulose-Based Hydrogels for Tissue Engineering Applications: A Brief Review. Polymers.

[48] Rastogi P., Kandasubramanian B. (2019). Review of Alginate-Based Hydrogel Bioprinting for Application in Tissue Engineering. Biofabrication.

[49] Badhe R.V., Godse A., Ahinkar A. (2020). Biomaterials in 3D Printing: A Special Emphasis on Nanocellulose. Indian J. Pharm. Educ. Res..

[50] Cui X., Li J., Hartanto Y., Durham M., Tang J., Zhang H., Hooper G., Lim K., Woodfield T. (2020). Advances in Extrusion 3D Bioprinting: A Focus on Multicomponent Hydrogel-Based Bioinks. Adv. Healthc. Mater..

[51] Mancha Sánchez E., Gómez-Blanco J.C., López Nieto E., Casado J.G., Macías-García A., Díaz Díez M.A., Carrasco-Amador J.P., Torrejón Martín D., Sánchez-Margallo F.M., Pagador J.B. (2020). Hydrogels for Bioprinting: A Systematic Review of Hydrogels Synthesis, Bioprinting Parameters, and Bioprinted Structures Behavior. Front. Bioeng. Biotechnol..

[52] Pahlevanzadeh F., Mokhtari H., Bakhsheshi-Rad H.R., Emadi R., Kharaziha M., Valiani A., Poursamar S.A., Ismail A.F., RamaKrishna S., Berto F. (2020). Recent Trends in Three-Dimensional Bioinks Based on Alginate for Biomedical Applications. Materials.

[53] Venugopal V. (2019). Sulfated and Non-Sulfated Polysaccharides from Seaweeds and Their Uses: An Overview. EC Nutr..

[54] Arokiarajan M.S., Thirunavukkarasu R., Joseph J., Ekaterina O., Aruni W. (2022). Advance Research in Biomedical Applications on Marine Sulfated Polysaccharide. Int. J. Biol. Macromol..

[55] Luo Y., Li Y., Qin X., Wa Q. (2018). 3D Printing of Concentrated Alginate/Gelatin Scaffolds with Homogeneous Nano Apatite Coating for Bone Tissue Engineering. Mater. Des..

[56] Yang X., Lu Z., Wu H., Li W., Zheng L., Zhao J. (2018). Collagen-Alginate as Bioink for Three-Dimensional (3D) Cell Printing Based Cartilage Tissue Engineering. Mater. Sci. Eng. C.

[57] Somasekharan L.T., Raju R., Kumar S., Geevarghese R., Nair R.P., Kasoju N., Bhatt A. (2021). Biofabrication of Skin Tissue Constructs Using Alginate, Gelatin and Diethylaminoethyl Cellulose Bioink. Int. J. Biol. Macromol..

[58] Schmid R., Schmidt S.K., Detsch R., Horder H., Blunk T., Schrüfer S., Schubert D.W., Fischer L., Thievessen I., Heltmann-Meyer S. (2022). A New Printable Alginate/Hyaluronic Acid/Gelatin Hydrogel Suitable for Biofabrication of In Vitro and In Vivo Metastatic Melanoma Models. Adv. Funct. Mater..

[59] Lewicki J., Bergman J., Kerins C., Hermanson O. (2019). Optimization of 3D Bioprinting of Human Neuroblastoma Cells Using Sodium Alginate Hydrogel. Bioprinting.

[60] Sarker M.D., Naghieh S., McInnes A.D., Ning L., Schreyer D.J., Chen X. (2019). Bio-Fabrication of Peptide-Modified Alginate Scaffolds: Printability, Mechanical Stability and Neurite Outgrowth Assessments. Bioprinting.

[61] Li H., Li N., Zhang H., Zhang Y., Suo H., Wang L., Xu M. (2020). Three-Dimensional Bioprinting of Perfusable Hierarchical Microchannels with Alginate and Silk Fibroin Double Cross-Linked Network. 3D Print. Addit. Manuf..

[62] Li L., Qin S., Peng J., Chen A., Nie Y., Liu T., Song K. (2020). Engineering Gelatin-Based Alginate/Carbon Nanotubes Blend Bioink for Direct 3D Printing of Vessel Constructs. Int. J. Biol. Macromol..

[63] Liu S., Zhang H., Hu Q., Shen Z., Rana D., Ramalingam M. (2020). Designing Vascular Supportive Albumen-Rich Composite Bioink for Organ 3D Printing. J. Mech. Behav. Biomed. Mater..

[64] Delkash Y., Gouin M., Rimbeault T., Mohabatpour F., Papagerakis P., Maw S., Chen X. (2021). Bioprinting and In Vitro Characterization of an Eggwhite-Based Cell-Laden Patch for Endothelialized Tissue Engineering Applications. J. Funct. Biomater..

[65] Dogan L., Scheuring R., Wagner N., Ueda Y., Schmidt S., Wörsdörfer P., Groll J., Ergün S. (2021). Human IPSC-Derived Mesodermal Progenitor Cells Preserve Their Vasculogenesis Potential after Extrusion and Form Hierarchically Organized Blood Vessels. Biofabrication.

[66] Kim E., Seok J.M., Bin Bae S., Park S.A., Park W.H. (2021). Silk Fibroin Enhances Cytocompatibilty and Dimensional Stability of Alginate Hydrogels for Light-Based Three-Dimensional Bioprinting. Biomacromolecules.

[67] Mihaila S.M., Gaharwar A.K., Reis R.L., Marques A.P., Gomes M.E., Khademhosseini A. (2013). Photocrosslinkable Kappa-Carrageenan Hydrogels for Tissue Engineering Applications. Adv. Healthc. Mater..

[68] Wilson S.A., Cross L.M., Peak C.W., Gaharwar A.K. (2017). Shear-Thinning and Thermo-Reversible Nanoengineered Inks for 3D Bioprinting. ACS Appl. Mater. Interfaces.

[69] Chimene D., Peak C.W., Gentry J.L., Carrow J.K., Cross L.M., Mondragon E., Cardoso G.B., Kaunas R., Gaharwar A.K. (2018). Nanoengineered Ionic–Covalent Entanglement (NICE) Bioinks for 3D Bioprinting. ACS Appl. Mater. Interfaces.

[70] Li H., Tan Y.J., Liu S., Li L. (2018). Three-Dimensional Bioprinting of Oppositely Charged Hydrogels with Super Strong Interface Bonding. ACS Appl. Mater. Interfaces.

[71] Li H., Tan Y.J., Li L. (2018). A Strategy for Strong Interface Bonding by 3D Bioprinting of Oppositely Charged κ-Carrageenan and Gelatin Hydrogels. Carbohydr. Polym..

[72] Tytgat L., Van Damme L., del Pilar Ortega Arevalo M., Declercq H., Thienpont H., Otteveare H., Blondeel P., Dubruel P., Van Vlierberghe S. (2019). Extrusion-Based 3D Printing of Photo-Crosslinkable Gelatin and κ-Carrageenan Hydrogel Blends for Adipose Tissue Regeneration. Int. J. Biol. Macromol..

[73] Kim M.H., Lee Y.W., Jung W.-K., Oh J., Nam S.Y. (2019). Enhanced Rheological Behaviors of Alginate Hydrogels with Carrageenan for Extrusion-Based Bioprinting. J. Mech. Behav. Biomed. Mater..

[74] Chimene D., Miller L., Cross L.M., Jaiswal M.K., Singh I., Gaharwar A.K. (2020). Nanoengineered Osteoinductive Bioink for 3D Bioprinting Bone Tissue. ACS Appl. Mater. Interfaces.

[75] López-Marcial G.R., Zeng A.Y., Osuna C., Dennis J., García J.M., O’Connell G.D. (2018). Agarose-Based Hydrogels as Suitable Bioprinting Materials for Tissue Engineering. ACS Biomater. Sci. Eng..

[76] Senior J.J., Cooke M.E., Grover L.M., Smith A.M. (2019). Fabrication of Complex Hydrogel Structures Using Suspended Layer Additive Manufacturing (SLAM). Adv. Funct. Mater..

[77] Cidonio G., Cooke M., Glinka M., Dawson J.I., Grover L., Oreffo R.O.C. (2019). Printing Bone in a Gel: Using Nanocomposite Bioink to Print Functionalised Bone Scaffolds. Mater. Today Bio.

[78] Aydin L., Kucuk S., Kenar H. (2020). A Universal Self-eroding Sacrificial Bioink That Enables Bioprinting at Room Temperature. Polym. Adv. Technol..

[79] Butler H.M., Naseri E., MacDonald D.S., Tasker R.A., Ahmadi A. (2021). Investigation of Rheology, Printability, and Biocompatibility of N,O-Carboxymethyl Chitosan and Agarose Bioinks for 3D Bioprinting of Neuron Cells. Materialia.

[80] Kothari D., Das D., Patel S., Goyal A., Tripura W., Informatics M., Diego S., Ramawat K.G., Mérillon J.-M. (2021). Polysaccharides.

[81] Choe G., Park J., Park H., Lee J. (2018). Hydrogel Biomaterials for Stem Cell Microencapsulation. Polymers.

[82] Lee H.-R., Jung S.M., Yoon S., Yoon W.H., Park T.H., Kim S., Shin H.W., Hwang D.S., Jung S. (2019). Immobilization of Planktonic Algal Spores by Inkjet Printing. Sci. Rep..

[83] Bociaga D., Bartniak M., Grabarczyk J., Przybyszewska K. (2019). Sodium Alginate/Gelatine Hydrogels for Direct Bioprinting—The Effect of Composition Selection and Applied Solvents on the Bioink Properties. Materials.

[84] Huettner N., Dargaville T.R., Forget A. (2018). Discovering Cell-Adhesion Peptides in Tissue Engineering: Beyond RGD. Trends Biotechnol..

[85] Datta P., Ayan B., Ozbolat I.T. (2017). Bioprinting for Vascular and Vascularized Tissue Biofabrication. Acta Biomater..

[86] Campo V.L., Kawano D.F., da Silva D.B., Carvalho I. (2009). Carrageenans: Biological Properties, Chemical Modifications and Structural Analysis – A Review. Carbohydr. Polym..

[87] Geonzon L.C., Descallar F.B.A., Du L., Bacabac R.G., Matsukawa S. (2020). Gelation Mechanism and Network Structure in Gels of Carrageenans and Their Mixtures Viewed at Different Length Scales – A Review. Food Hydrocoll..

[88] Yegappan R., Selvaprithiviraj V., Amirthalingam S., Jayakumar R. (2018). Carrageenan Based Hydrogels for Drug Delivery, Tissue Engineering and Wound Healing. Carbohydr. Polym..

[89] Jafari A., Farahani M., Sedighi M., Rabiee N., Savoji H. (2022). Carrageenans for Tissue Engineering and Regenerative Medicine Applications: A Review. Carbohydr. Polym..

[90] KapMA. https://www.adbioink.com/product/kappa-carrageenan-bioink/.

[91] Zarrintaj P., Manouchehri S., Ahmadi Z., Saeb M.R., Urbanska A.M., Kaplan D.L., Mozafari M. (2018). Agarose-Based Biomaterials for Tissue Engineering. Carbohydr. Polym..

[92] Graham S., Marina P.F., Blencowe A. (2019). Thermoresponsive Polysaccharides and Their Thermoreversible Physical Hydrogel Networks. Carbohydr. Polym..

[93] Pokusaev B., Vyazmin A., Zakharov N., Karlov S., Nekrasov D., Reznik V., Khramtsov D. (2020). Thermokinetics and Rheology of Agarose Gel Applied to Bioprinting Technology. Therm. Sci..

[94] Topuz F., Nadernezhad A., Caliskan O.S., Menceloglu Y.Z., Koc B. (2018). Nanosilicate Embedded Agarose Hydrogels with Improved Bioactivity. Carbohydr. Polym..

[95] Heidari H., Taylor H. (2020). Multilayered Microcasting of Agarose–Collagen Composites for Neurovascular Modeling. Bioprinting.

[96] BeMiller J.N. (2019). Polysaccharides. Carbohydrate Chemistry for Food Scientists.

[97] Banerjee S., Bhattacharya S. (2012). Food Gels: Gelling Process and New Applications. Crit. Rev. Food Sci. Nutr..

[98] Munarin F., Tanzi M.C., Petrini P. (2012). Advances in Biomedical Applications of Pectin Gels. Int. J. Biol. Macromol..

[99] Heidarian P., Kouzani A.Z., Kaynak A., Paulino M., Nasri-Nasrabadi B., Zolfagharian A., Varley R. (2020). Dynamic Plant-Derived Polysaccharide-Based Hydrogels. Carbohydr. Polym..

[100] Habib A., Sathish V., Mallik S., Khoda B. (2018). 3D Printability of Alginate-Carboxymethyl Cellulose Hydrogel. Materials.

[101] Hodder E., Duin S., Kilian D., Ahlfeld T., Seidel J., Nachtigall C., Bush P., Covill D., Gelinsky M., Lode A. (2019). Investigating the Effect of Sterilisation Methods on the Physical Properties and Cytocompatibility of Methyl Cellulose Used in Combination with Alginate for 3D-Bioplotting of Chondrocytes. J. Mater. Sci. Mater. Med..

[102] Im S., Choe G., Seok J.M., Yeo S.J., Lee J.H., Kim W.D., Lee J.Y., Park S.A. (2022). An Osteogenic Bioink Composed of Alginate, Cellulose Nanofibrils, and Polydopamine Nanoparticles for 3D Bioprinting and Bone Tissue Engineering. Int. J. Biol. Macromol..

[103] Ronzoni F.L., Aliberti F., Scocozza F., Benedetti L., Auricchio F., Sampaolesi M., Cusella G., Redwan I.N., Ceccarelli G., Conti M. (2022). Myoblast 3D Bioprinting to Burst in Vitro Skeletal Muscle Differentiation. J. Tissue Eng. Regen. Med..

[104] Mendes B.B., Gómez-Florit M., Hamilton A.G., Detamore M.S., Domingues R.M.A., Reis R.L., Gomes M.E. (2019). Human Platelet Lysate-Based Nanocomposite Bioink for Bioprinting Hierarchical Fibrillar Structures. Biofabrication.

[105] Maturavongsadit P., Narayanan L.K., Chansoria P., Shirwaiker R., Benhabbour S.R. (2021). Cell-Laden Nanocellulose/Chitosan-Based Bioinks for 3D Bioprinting and Enhanced Osteogenic Cell Differentiation. ACS Appl. Bio Mater..

[106] Boonlai W., Tantishaiyakul V., Hirun N. (2022). Characterization of Κ-carrageenan/Methylcellulose/Cellulose Nanocrystal Hydrogels for 3D Bioprinting. Polym. Int..

[107] Wu Z., Xie S., Kang Y., Shan X., Li Q., Cai Z. (2021). Biocompatibility Evaluation of a 3D-Bioprinted Alginate-GelMA-Bacteria Nanocellulose (BNC) Scaffold Laden with Oriented-Growth RSC96 Cells. Mater. Sci. Eng. C.

[108] Das R., Lee C.P., Prakash A., Hashimoto M., Fernandez J.G. (2022). Geometrical Control of Degradation and Cell Delivery in 3D Printed Nanocellulose Hydrogels. Mater. Today Commun..

[109] García-Lizarribar A., Fernández-Garibay X., Velasco-Mallorquí F., Castaño A.G., Samitier J., Ramon-Azcon J. (2018). Composite Biomaterials as Long-Lasting Scaffolds for 3D Bioprinting of Highly Aligned Muscle Tissue. Macromol. Biosci..

[110] Fan Y., Yue Z., Lucarelli E., Wallace G.G. (2020). Hybrid Printing Using Cellulose Nanocrystals Reinforced GelMA/HAMA Hydrogels for Improved Structural Integration. Adv. Healthc. Mater..

[111] Ji S., Abaci A., Morrison T., Gramlich W.M., Guvendiren M. (2020). Novel Bioinks from UV-Responsive Norbornene-Functionalized Carboxymethyl Cellulose Macromers. Bioprinting.

[112] Li X., Deng Q., Zhuang T., Lu Y., Liu T., Zhao W., Lin B., Luo Y., Zhang X. (2020). 3D Bioprinted Breast Tumor Model for Structure–Activity Relationship Study. Bio-Design Manuf..

[113] Montheil T., Maumus M., Valot L., Lebrun A., Martinez J., Amblard M., Noël D., Mehdi A., Subra G. (2020). Inorganic Sol–Gel Polymerization for Hydrogel Bioprinting. ACS Omega.

[114] Ni T., Liu M., Zhang Y., Cao Y., Pei R. (2020). 3D Bioprinting of Bone Marrow Mesenchymal Stem Cell-Laden Silk Fibroin Double Network Scaffolds for Cartilage Tissue Repair. Bioconjug. Chem..

[115] Trachsel L., Johnbosco C., Lang T., Benetti E.M., Zenobi-Wong M. (2019). Double-Network Hydrogels Including Enzymatically Crosslinked Poly-(2-Alkyl-2-Oxazoline)s for 3D Bioprinting of Cartilage-Engineering Constructs. Biomacromolecules.

[116] Gantumur E., Nakahata M., Kojima M., Sakai S. (2020). Extrusion-Based Bioprinting through Glucose-Mediated Enzymatic Hydrogelation. Int. J. Bioprinting.

[117] Zidarič T., Milojević M., Gradišnik L., Stana Kleinschek K., Maver U., Maver T. (2020). Polysaccharide-Based Bioink Formulation for 3D Bioprinting of an In Vitro Model of the Human Dermis. Nanomaterials.

[118] Lan X., Ma Z., Szojka A.R.A., Kunze M., Mulet-Sierra A., Vyhlidal M.J., Boluk Y., Adesida A.B. (2021). TEMPO-Oxidized Cellulose Nanofiber-Alginate Hydrogel as a Bioink for Human Meniscus Tissue Engineering. Front. Bioeng. Biotechnol..

[119] Pereira R.F., Sousa A., Barrias C.C., Bártolo P.J., Granja P.L. (2018). A Single-Component Hydrogel Bioink for Bioprinting of Bioengineered 3D Constructs for Dermal Tissue Engineering. Mater. Horizons.

[120] Hu S., Martinez-Garcia F.D., Moeun B.N., Burgess J.K., Harmsen M.C., Hoesli C., de Vos P. (2021). An Immune Regulatory 3D-Printed Alginate-Pectin Construct for Immunoisolation of Insulin Producing β-Cells. Mater. Sci. Eng. C.

[121] Klemm D., Heublein B., Fink H., Bohn A. (2005). Cellulose: Fascinating Biopolymer and Sustainable Raw Material. Angew. Chemie Int. Ed..

[122] Vilela C., Pinto R.J.B., Figueiredo A.R.P., Neto C.P., Silvestre A.J.D., Freire C.S.R. (2017). Development and Applications of Cellulose Nanofibres Based Polymer Nanocomposites. Advanced Composite Materials: Properties and Applications.

[123] Li Y.-Y., Wang B., Ma M.-G., Wang B. (2018). Review of Recent Development on Preparation, Properties, and Applications of Cellulose-Based Functional Materials. Int. J. Polym. Sci..

[124] Hon D.N.-S. (2001). Cellulose: Chemistry and Technology. Encyclopedia of Materials: Science and Technology.

[125] Jedvert K., Heinze T. (2017). Cellulose Modification and Shaping—A Review. J. Polym. Eng..

[126] Zennifer A., Senthilvelan P., Sethuraman S., Sundaramurthi D. (2021). Key Advances of Carboxymethyl Cellulose in Tissue Engineering & 3D Bioprinting Applications. Carbohydr. Polym..

[127] Mallakpour S., Tukhani M., Hussain C.M. (2021). Recent Advancements in 3D Bioprinting Technology of Carboxymethyl Cellulose-Based Hydrogels: Utilization in Tissue Engineering. Adv. Colloid Interface Sci..

[128] Dufresne A. (2012). Preparation of Microfibrillated Cellulose. Nanocellulose: From Nature to High Performance Tailored Materials.

[129] Klemm D., Kramer F., Moritz S., Lindström T., Ankerfors M., Gray D., Dorris A. (2011). Nanocelluloses: A New Family of Nature-Based Materials. Angew. Chemie Int. Ed..

[130] Curvello R., Raghuwanshi V.S., Garnier G. (2019). Engineering Nanocellulose Hydrogels for Biomedical Applications. Adv. Colloid Interface Sci..

[131] Wang Q., Sun J., Yao Q., Ji C., Liu J., Zhu Q. (2018). 3D Printing with Cellulose Materials. Cellulose.

[132] CELLINK Bioink. https://www.cellink.com/product/cellink-bioink/.

[133] Wu Y., Lin Z.Y., Wenger A.C., Tam K.C., Tang X. (2018). 3D Bioprinting of Liver-Mimetic Construct with Alginate/Cellulose Nanocrystal Hybrid Bioink. Bioprinting.

[134] Flutto L. (2003). PECTIN Properties and Determination. Encyclopedia of Food Sciences and Nutrition.

[135] Narasimman P., Sethuraman P. (2016). An Overview on the Fundamentals of Pectin. Int. J. Adv. Res..

[136] Willats W.G.T., Knox J.P., Mikkelsen J.D. (2006). Pectin: New Insights into an Old Polymer Are Starting to Gel. Trends Food Sci. Technol..

[137] Mishra R.K., Banthia A.K., Majeed A.B.A. (2012). Pectin Based Formulations for Biomedical Applications: A Review. Asian J. Pharm. Clin. Res..

[138] Li D., Li J., Dong H., Li X., Zhang J., Ramaswamy S., Xu F. (2021). Pectin in Biomedical and Drug Delivery Applications: A Review. Int. J. Biol. Macromol..

[139] Jovic T.H., Kungwengwe G., Mills A.C., Whitaker I.S. (2019). Plant-Derived Biomaterials: A Review of 3D Bioprinting and Biomedical Applications. Front. Mech. Eng..

[140] Indurkar A., Pandit A., Jain R., Dandekar P. (2021). Plant-Based Biomaterials in Tissue Engineering. Bioprinting.

[141] Tester R.F., Karkalas J., Qi X. (2004). Starch—Composition, Fine Structure and Architecture. J. Cereal Sci..

[142] Bean S.R., Zhu L., Smith B.M., Wilson J.D., Ioerger B.P., Tilley M. (2019). Starch and Protein Chemistry and Functional Properties. Sorghum and Millets.

[143] Gopinath V., Saravanan S., Al-Maleki A.R., Ramesh M., Vadivelu J. (2018). A Review of Natural Polysaccharides for Drug Delivery Applications: Special Focus on Cellulose, Starch and Glycogen. Biomed. Pharmacother..

[144] Aljohani W., Ullah M.W., Zhang X., Yang G. (2018). Bioprinting and Its Applications in Tissue Engineering and Regenerative Medicine. Int. J. Biol. Macromol..

[145] Carrow J.K., Kerativitayanan P., Jaiswal M.K., Lokhande G., Gaharwar A.K. (2015). Polymers for Bioprinting. Essentials of 3D Biofabrication and Translation.

[146] Maniglia B.C., Lima D.C., Matta Junior M.D., Le-Bail P., Le-Bail A., Augusto P.E.D. (2019). Hydrogels Based on Ozonated Cassava Starch: Effect of Ozone Processing and Gelatinization Conditions on Enhancing 3D-Printing Applications. Int. J. Biol. Macromol..

[147] Noè C., Tonda-Turo C., Chiappone A., Sangermano M., Hakkarainen M. (2020). Light Processable Starch Hydrogels. Polymers.

[148] Zhang L., Zheng T., Wu L., Han Q., Chen S., Kong Y., Li G., Ma L., Wu H., Zhao Y. (2021). Fabrication and Characterization of 3D-Printed Gellan Gum/Starch Composite Scaffold for Schwann Cells Growth. Nanotechnol. Rev..

[149] Chaisuwan W., Jantanasakulwong K., Wangtueai S., Phimolsiripol Y., Chaiyaso T., Techapun C., Phongthai S., You S., Regenstein J.M., Seesuriyachan P. (2020). Microbial Exopolysaccharides for Immune Enhancement: Fermentation, Modifications and Bioactivities. Food Biosci..

[150] Busuioc M., Mackiewicz K., Buttaro B.A., Piggot P.J. (2009). Role of Intracellular Polysaccharide in Persistence of Streptococcus Mutans. J. Bacteriol..

[151] Turner P.R., Murray E., McAdam C.J., McConnell M.A., Cabral J.D. (2020). Peptide Chitosan/Dextran Core/Shell Vascularized 3D Constructs for Wound Healing. ACS Appl. Mater. Interfaces.

[152] Lim W., Shin S.Y., Cha J.M., Bae H. (2021). Optimization of Polysaccharide Hydrocolloid for the Development of Bioink with High Printability/Biocompatibility for Coextrusion 3D Bioprinting. Polymers.

[153] Kapr J., Petersilie L., Distler T., Lauria I., Bendt F., Sauter C.M., Boccaccini A.R., Rose C.R., Fritsche E. (2021). Human Induced Pluripotent Stem Cell-Derived Neural Progenitor Cells Produce Distinct Neural 3D In Vitro Models Depending on Alginate/Gellan Gum/Laminin Hydrogel Blend Properties. Adv. Healthc. Mater..

[154] Piola B., Sabbatini M., Gino S., Invernizzi M., Renò F. (2022). 3D Bioprinting of Gelatin–Xanthan Gum Composite Hydrogels for Growth of Human Skin Cells. Int. J. Mol. Sci..

[155] Muthusamy S., Kannan S., Lee M., Sanjairaj V., Lu W.F., Fuh J.Y.H., Sriram G., Cao T. (2021). 3D Bioprinting and Microscale Organization of Vascularized Tissue Constructs Using Collagen-based Bioink. Biotechnol. Bioeng..

[156] Levato R., Visser J., Planell J.A., Engel E., Malda J., Mateos-Timoneda M.A. (2014). Biofabrication of Tissue Constructs by 3D Bioprinting of Cell-Laden Microcarriers. Biofabrication.

[157] Lozano R., Stevens L., Thompson B.C., Gilmore K.J., Gorkin R., Stewart E.M., Panhuis M., Romero-Ortega M., Wallace G.G. (2015). 3D Printing of Layered Brain-like Structures Using Peptide Modified Gellan Gum Substrates. Biomaterials.

[158] Hu D., Wu D., Huang L., Jiao Y., Li L., Lu L., Zhou C. (2018). 3D Bioprinting of Cell-Laden Scaffolds for Intervertebral Disc Regeneration. Mater. Lett..

[159] Wu D., Yu Y., Tan J., Huang L., Luo B., Lu L., Zhou C. (2018). 3D Bioprinting of Gellan Gum and Poly (Ethylene Glycol) Diacrylate Based Hydrogels to Produce Human-Scale Constructs with High-Fidelity. Mater. Des..

[160] Zhuang P., Ng W.L., An J., Chua C.K., Tan L.P. (2019). Layer-by-Layer Ultraviolet Assisted Extrusion-Based (UAE) Bioprinting of Hydrogel Constructs with High Aspect Ratio for Soft Tissue Engineering Applications. PLoS ONE.

[161] Chen Y., Xiong X., Liu X., Cui R., Wang C., Zhao G., Zhi W., Lu M., Duan K., Weng J. (2020). 3D Bioprinting of Shear-Thinning Hybrid Bioinks with Excellent Bioactivity Derived from Gellan/Alginate and Thixotropic Magnesium Phosphate-Based Gels. J. Mater. Chem. B.

[162] Costa J.B., Park J., Jorgensen A.M., Silva-Correia J., Reis R.L., Oliveira J.M., Atala A., Yoo J.J., Lee S.J. (2020). 3D Bioprinted Highly Elastic Hybrid Constructs for Advanced Fibrocartilaginous Tissue Regeneration. Chem. Mater..

[163] Naessens M., Cerdobbel A., Soetaert W., Vandamme E.J. (2005). Leuconostoc Dextransucrase and Dextran: Production, Properties and Applications. J. Chem. Technol. Biotechnol..

[164] Díaz-Montes E. (2021). Dextran: Sources, Structures, and Properties. Polysaccharides.

[165] Ng J.Y., Obuobi S., Chua M.L., Zhang C., Hong S., Kumar Y., Gokhale R., Ee P.L.R. (2020). Biomimicry of Microbial Polysaccharide Hydrogels for Tissue Engineering and Regenerative Medicine – A Review. Carbohydr. Polym..

[166] García-Ochoa F., Santos V., Casas J., Gómez E. (2000). Xanthan Gum: Production, Recovery, and Properties. Biotechnol. Adv..

[167] Patel J., Maji B., Moorthy N.S.H.N., Maiti S. (2020). Xanthan Gum Derivatives: Review of Synthesis, Properties and Diverse Applications. RSC Adv..

[168] Kumar A., Rao K.M., Han S.S. (2018). Application of Xanthan Gum as Polysaccharide in Tissue Engineering: A Review. Carbohydr. Polym..

[169] GelXG. https://www.cellink.com/product/gelxg/.

[170] GelXA. https://www.cellink.com/product/gelxa/.

[171] Giavasis I., Harvey L.M., McNeil B. (2000). Gellan Gum. Crit. Rev. Biotechnol..

[172] Bajaj I.B., Survase S.A., Saudagar P.S., Singhal R.S. (2007). Gellan Gum: Fermentative Production, Downstream Processing and Applications. Food Technol. Biotechnol..

[173] Stevens L.R., Gilmore K.J., Wallace G.G., Panhuis M. (2016). Tissue Engineering with Gellan Gum. Biomater. Sci..

[174] GumMA. https://www.adbioink.com/product/gellan-gum-bioink/.

[175] Cheng K.-C., Demirci A., Catchmark J.M. (2011). Pullulan: Biosynthesis, Production, and Applications. Appl. Microbiol. Biotechnol..

[176] Singh R.S., Saini G.K., Kennedy J.F. (2008). Pullulan: Microbial Sources, Production and Applications. Carbohydr. Polym..

[177] Qi X., Su T., Zhang M., Tong X., Pan W., Zeng Q., Shen J. (2020). Sustainable, Flexible and Biocompatible Hydrogels Derived from Microbial Polysaccharides with Tailorable Structures for Tissue Engineering. Carbohydr. Polym..

[178] Della Giustina G., Gandin A., Brigo L., Panciera T., Giulitti S., Sgarbossa P., D’Alessandro D., Trombi L., Danti S., Brusatin G. (2019). Polysaccharide Hydrogels for Multiscale 3D Printing of Pullulan Scaffolds. Mater. Des..

[179] Rinaudo M. (2006). Chitin and Chitosan: Properties and Applications. Prog. Polym. Sci..

[180] Tokura S., Tamura H. (2007). Chitin and Chitosan. Comprehensive Glycoscience.

[181] Parhi R. (2020). Drug Delivery Applications of Chitin and Chitosan: A Review. Environ. Chem. Lett..

[182] Tao F., Cheng Y., Shi X., Zheng H., Du Y., Xiang W., Deng H. (2020). Applications of Chitin and Chitosan Nanofibers in Bone Regenerative Engineering. Carbohydr. Polym..

[183] Li Y., Jiang X., Li L., Chen Z.-N., Gao G., Yao R., Sun W. (2018). 3D Printing Human Induced Pluripotent Stem Cells with Novel Hydroxypropyl Chitin Bioink: Scalable Expansion and Uniform Aggregation. Biofabrication.

[184] Kołodziejska M., Jankowska K., Klak M., Wszoła M. (2021). Chitosan as an Underrated Polymer in Modern Tissue Engineering. Nanomaterials.

[185] Ahmadi F., Oveisi Z., Samani S.M., Amoozgar Z. (2015). Chitosan Based Hydrogels: Characteristics and Pharmaceutical Applications. Res. Pharm. Sci..

[186] Li J., Cai C., Li J., Li J., Li T., Sun T., Wang L., Wu H., Yu G. (2018). Chitosan-Based Nanomaterials for Drug Delivery. Molecules.

[187] Deng A., Yang Y., Du S., Yang X., Pang S., Wang X., Yang S. (2021). Preparation of a Recombinant Collagen-Peptide (RHC)-Conjugated Chitosan Thermosensitive Hydrogel for Wound Healing. Mater. Sci. Eng. C.

[188] Feng P., Luo Y., Ke C., Qiu H., Wang W., Zhu Y., Hou R., Xu L., Wu S. (2021). Chitosan-Based Functional Materials for Skin Wound Repair: Mechanisms and Applications. Front. Bioeng. Biotechnol..

[189] Chitoink. https://www.cellink.com/product/chitoink/.

[190] Bergonzi C., Di Natale A., Zimetti F., Marchi C., Bianchera A., Bernini F., Silvestri M., Bettini R., Elviri L. (2019). Study of 3D-Printed Chitosan Scaffold Features after Different Post-Printing Gelation Processes. Sci. Rep..

[191] Ku J., Seonwoo H., Park S., Jang K.-J., Lee J., Lee M., Lim J.W., Kim J., Chung J.H. (2020). Cell-Laden Thermosensitive Chitosan Hydrogel Bioinks for 3D Bioprinting Applications. Appl. Sci..

[192] Shen Y., Tang H., Huang X., Hang R., Zhang X., Wang Y., Yao X. (2020). DLP Printing Photocurable Chitosan to Build Bio-Constructs for Tissue Engineering. Carbohydr. Polym..

[193] Puertas-Bartolomé M., Włodarczyk-Biegun M.K., del Campo A., Vázquez-Lasa B., San Román J. (2020). 3D Printing of a Reactive Hydrogel Bio-Ink Using a Static Mixing Tool. Polymers.

[194] Magli S., Rossi G.B., Risi G., Bertini S., Cosentino C., Crippa L., Ballarini E., Cavaletti G., Piazza L., Masseroni E. (2020). Design and Synthesis of Chitosan—Gelatin Hybrid Hydrogels for 3D Printable in Vitro Models. Front. Chem..

[195] Hu T., Cui X., Zhu M., Wu M., Tian Y., Yao B., Song W., Niu Z., Huang S., Fu X. (2020). 3D-Printable Supramolecular Hydrogels with Shear-Thinning Property: Fabricating Strength Tunable Bioink via Dual Crosslinking. Bioact. Mater..

[196] Pisani S., Dorati R., Scocozza F., Mariotti C., Chiesa E., Bruni G., Genta I., Auricchio F., Conti M., Conti B. (2020). Preliminary Investigation on a New Natural Based Poly(Gamma-Glutamic Acid)/Chitosan Bioink. J. Biomed. Mater. Res. Part B Appl. Biomater..

[197] Tonda-Turo C., Carmagnola I., Chiappone A., Feng Z., Ciardelli G., Hakkarainen M., Sangermano M. (2020). Photocurable Chitosan as Bioink for Cellularized Therapies towards Personalized Scaffold Architecture. Bioprinting.

[198] Sze J.H., Brownlie J.C., Love C.A. (2016). Biotechnological Production of Hyaluronic Acid: A Mini Review. 3 Biotech.

[199] Badri A., Williams A., Linhardt R.J., Koffas M.A. (2018). The Road to Animal-Free Glycosaminoglycan Production: Current Efforts and Bottlenecks. Curr. Opin. Biotechnol..

[200] Celikkin N., Rinoldi C., Costantini M., Trombetta M., Rainer A., Święszkowski W. (2017). Naturally Derived Proteins and Glycosaminoglycan Scaffolds for Tissue Engineering Applications. Mater. Sci. Eng. C.

[201] Sodhi H., Panitch A. (2020). Glycosaminoglycans in Tissue Engineering: A Review. Biomolecules.

[202] Dovedytis M., Liu Z.J., Bartlett S. (2020). Hyaluronic Acid and Its Biomedical Applications: A Review. Eng. Regen..

[203] Lee S.J., Seok J.M., Lee J.H., Lee J., Kim W.D., Park S.A. (2021). Three-Dimensional Printable Hydrogel Using a Hyaluronic Acid/Sodium Alginate Bio-Ink. Polymers.

[204] Ma L., Li Y., Wu Y., Yu M., Aazmi A., Gao L., Xue Q., Luo Y., Zhou H., Zhang B. (2020). 3D Bioprinted Hyaluronic Acid-Based Cell-Laden Scaffold for Brain Microenvironment Simulation. Bio-Design Manuf..

[205] Hauptstein J., Forster L., Nadernezhad A., Horder H., Stahlhut P., Groll J., Blunk T., Teßmar J. (2022). Bioink Platform Utilizing Dual-Stage Crosslinking of Hyaluronic Acid Tailored for Chondrogenic Differentiation of Mesenchymal Stromal Cells. Macromol. Biosci..

[206] Hauptstein J., Forster L., Nadernezhad A., Groll J., Teßmar J., Blunk T. (2022). Tethered TGF-Β1 in a Hyaluronic Acid-Based Bioink for Bioprinting Cartilaginous Tissues. Int. J. Mol. Sci..

[207] PhotoHA^TM^-IRG. https://www.sigmaaldrich.com/PT/en/product/aldrich/917079.

[208] Hölzl K., Lin S., Tytgat L., Van Vlierberghe S., Gu L., Ovsianikov A. (2016). Bioink Properties before, during and after 3D Bioprinting. Biofabrication.

